# ﻿Nineteen new species of *Desmopachria* Babington, 1841 (Coleoptera, Adephaga, Dytiscidae, Hydroporinae, Hyphydrini) with notes on the taxonomy of the genus

**DOI:** 10.3897/zookeys.1136.72744

**Published:** 2022-12-16

**Authors:** Kelly B. Miller

**Affiliations:** 1 Department of Biology and Museum of Southwestern Biology University of New Mexico, Albuquerque, NM 87131–0001, USA Department of Biology and Museum of Southwestern Biology University of New Mexico Albuquerque United States of America

**Keywords:** Diving water beetles, male genitalia, South America, taxonomy

## Abstract

Nineteen new species of *Desmopachria* Babington, 1841 are described from multiple species groups. Two new species groups are erected, the *Desmopachriaapicodente* species group and the *Desmopachriabifurcita* species group. *Desmopachriadivergens***sp. nov.** (Venezuela), *Desmopachrialineata***sp. nov.** (Venezuela), *Desmopachriasurinamensis***sp. nov.** (Suriname), and *Desmopachriatenua***sp. nov.** (Guyana) are described in *Desmopachria* but are not assigned to a species group. *Desmopachriaapicodente***sp. nov.** (Guyana, Venezuela), *Desmopachrialateralis***sp. nov.** (Venezuela), and *Desmopachriatumida***sp. nov.** (Venezuela) are described in the new *Desmopachriaapicodente* species group and are the only members of the group. *Desmopachriabifurcita***sp. nov.** (Peru), and *Desmopachrialata***sp. nov.** (Brazil) are described in the new *Desmopachriabifurcita* group. Other members of the *Desmopachriabifurcita* group are *Desmopachriabifasciata* Zimmermann, *Desmopachriabolivari* Miller, *Desmopachriaovalis* Sharp, and *Desmopachriavarians* (each previously “ungrouped”). *Desmopachriapseudocavia***sp. nov.** (Venezuela) is described in the *Desmopachriaconvexa-signata* species group. *Desmopachriawolfei***sp. nov.** (Venezuela) is described in the *Desmopachrianitida* species group. *Desmopachriaangulata***sp. nov.** (Guyana, Suriname), *Desmopachriaemarginata***sp. nov.** (Guyana, Suriname, Venezuela), *Desmopachriaimparis***sp. nov.** (Guyana), *Desmopachriaimpunctata***sp. nov.** (Suriname, Venezuela), and *Desmopachriatruncata***sp. nov.** (Guyana, Suriname) are described in the *Desmopachriaportmanni-aldessa* species group. *Desmopachriabisulcata***sp. nov.** (Suriname), and *Desmopachriairregulara***sp. nov.** (Venezuela) are described in the *Desmopachriaportmanni-portmanni* species group. *Desmopachriarobusta***sp. nov.** (Venezuela) is described in the *Desmopachriastriola* species group. A key to the species groups is included. Male genitalia are figured for all new species and dorsal habitus images are provided for most new species.

## ﻿Introduction

The hyperdiverse diving beetle (Dytiscidae) genus *Desmopachria* Babington includes approximately 133 species prior to this paper (Nilsson and Hájek 2019). Within *Desmopachria*, there are a number of groups that are relatively well defined by distinctive synapomorphies, many of which were previously regarded as subgenera (Guignot 1949; Young 1980). Later these groups were relegated to species-group status because of concerns about monophyly of several of them (Miller 2001). A rather large number of new species in these various groups have been described in the past few years (Miller 1999, 2001, 2005; Braga and Ferreira Jr 2011, 2014; Gustafson and Miller 2012; Makhan 2012, 2015; Megna and Sanchez-Fernandez 2014; Miller and Wolfe 2018). Given the regular description of new species, it can be expected that species discovery may continue apace for some time. Species appear to be abundant and narrowly endemic, and as increased collecting occurs in new areas, especially in under-collected areas of South America, new species can be expected to be found. Fortunately, it seems that species in the genus often have very distinctive male genitalia and other features that make their delimitation and diagnoses possible.

Even though several of the subgroups in *Desmopachria* (whether subgenera or species groups) have rather distinctive synapomorphies and are likely monophyletic, there are many species that are not currently well-placed into them, which is partly what led to the obliteration of the subgenera and the recognition of an “ungrouped” collection of species (Miller 2001). Some species in this collection may belong to one of the subgroups, but descriptions do not provide enough information for their placement and specimens (primary types) have not been examined. In other cases, modern authors have described species, but did not place them in groups nor adequately describe them such that they can be placed into groups (specifically Makhan 2012, 2015).

The objective of this paper is to describe 19 new species of *Desmopachria*. Some of these belong to existing groups, some are placed in newly proposed groups, and others are not clearly placed into any of these groups yet are clearly in *Desmopachria*, so are left ungrouped. Following recent previous treatments of *Desmopachria* (Miller 1999, 2001, 2005; Braga and Ferreira Jr 2010, 2011, 2014, 2018; Gustafson and Miller 2012; Megna and Sanchez-Fernandez 2014; Miller and Wolfe 2018), keys to species are not provided; instead, specimens should be keyed to species groups, then dissected and male genitalia and other diagnostic features compared with published illustrations and diagnoses of species. New records are also provided for some known species, and some comments are provided regarding several of the groups.

## ﻿Materials and methods

Methods closely follow recent work on the group by Miller and Wolfe (2018, 2019) and Miller (2020). Measurements were made with an ocular scale on a Zeiss Discovery V8 dissecting microscope. The diagnostic range of measurements of structures was emphasized, so the largest and smallest specimens were preferentially measured to the extent possible. In the case of available series of specimens ten or fewer, all intact specimens were measured. Specimens not intact were not measured. Measurements in the text are abbreviated as follows, while the ratios TL/GW and HW/EW were also calculated.

**TL** total length

**GW** greatest width across elytra

**PW** greatest width of pronotum

**HW** greatest width of head

**EW** distance between eyes

Illustrations were made using a drawing tube on a Zeiss Discovery V8 dissecting scope. Sketches were first done in pencil then scanned, placed into an Adobe Illustrator artboard, and inked digitally using vector lines.

Specimens of *Desmopachria* were examined from the following collections:

**CSBD**Center for Biological Diversity, University of Guyana.

**KBMC**Kelly B. Miller Collection, Museum of Southwestern Biology, University of New Mexico, Albuquerque, NM, USA.

**SEMC**University of Kansas Natural History Museum, University of Kansas, Lawrence, Kansas, USA (A.E.Z. Short).

**MIZA**Museo del Instituto de Zoología Agrícola Francisco Fernández Yépez, Universidad Central de Venezuela, Maracay, Venezuela (L. Joly).

**MSBA**Museum of Southwestern Biology Division of Arthropods, University of New Mexico, Albuquerque, NM, USA (K.B. Miller).

**NZCS**National Zoological Collection of Suriname, Paramaribo, Suriname (P. Ouboter).

**USNM**United States National Collection of Insects, Smithsonian Institution, Washington, DC, USA (C. Micheli).

### ﻿Key to the species groups of *Desmopachria*

**Table:** 

1	Anterior metatibial spine serrate	***Desmopachriavicina* group**
–	Anterior metatibial spine not serrate	**2**
2	Pronotum with an incised stria on each side of base	***Desmopachriadispersa* group**
–	Pronotum without basal striae	**3**
3	Prosternal process sexually dimorphic, male process apically strongly bifid, area between rami forming a deep pit, female process not as in male	***Desmopachriaportmanni* group**
–	Prosternal process not sexually dimorphic, not forked in either sex	**4**
4	Elytron with a distinct sutural stria (e.g., Fig. 72)	***Desmopachriastriola* group**
–	Elytron without a distinct sutural stria	**5**
5	Anterior clypeal margin sexually dimorphic, in males strongly modified, thin, translucent, strongly up-turned, anteriorly beaded in female, but not as strongly modified as in male	***Desmopachriaubangoides* group**
–	Anterior clypeal margin not sexually dimorphic, anteriorly beaded in both sexes	**6**
6	Male lateral lobes deeply bifid, apex divided into two elongate rami (e.g., Fig. 50)	***Desmopachrianitida* group**
–	Male lateral lobes not deeply bifid	**7**
7	Male lateral lobes with anteapical, articulable process (e.g., Fig. 45)	***Desmopachriaconvexa* group**
–	Male lateral lobes without anteapical, articulable process	**8**
8	Male median and lateral lobes very strongly robust and heavily sclerotized	***Desmopachriaglabricula* group**
–	Male median and lateral lobes not strongly robust and sclerotized	**9**
9	Male median lobe short and broad, much shorter than lateral lobes (e.g., Figs 29, 32, 35, 36, 39, 41), male lateral lobes elongate, dorsoventrally flattened and laterally broad, medially distinctly bent dorsad, apical portion flattened and straight (e.g., Figs 30, 33, 37, 42)	***Desmopachriabifurcita* group**
–	Male median and lateral lobes not as described above	**10**
10	Male lateral lobe apically with a distinct spur or tooth (e.g., Figs 17, 22, 27)	***Desmopachriaapicodente* group**
–	Male lateral lobe apically without distinct spur or tooth	**ungrouped *Desmopachria***

### ﻿Ungrouped *Desmopachria*

Many *Desmopachria* species do not have the distinctive synapomorphies of the various species groups recognized by Miller (2001) and in this work (see key above), or originally treated as subgenera (Young 1980). Most of these species were originally placed in Desmopachria (Desmopachria) Babington, but that subgenus does not exhibit a clear synapomorphy, instead it is a collection of species that do not have features present in other subgroups of *Desmopachria*. The following new species also do not fit into any of the defined species groups and are placed among the ungrouped *Desmopachria*.

#### ﻿ Desmopachria divergens sp. nov.

Taxon classificationAnimaliaColeopteraDytiscidae

B315FC3A-8513-56A5-80EF-E3130F847C99

http://zoobank.org/BEF901E2-265C-4401-8121-57E66ED970DF

Figures 1 , 2 , 76

##### Type locality.

Venezuela, Amazonas State, Communidad Caño Gato on Rio Sipapo, 4°58.838'N 67°44.341'W.

##### Diagnosis.

This species is characterized by dorsal iridescence and the shape of the male genitalia. The male median lobe in ventral aspect is extremely broad and apically very broadly truncate combined with lateral lobes with the apices strongly curved laterad and divergent (Fig. 2). This species is dorsally iridescent but lacks the bifurcate prosternal process of the *Desmopachriaportmanni* species group. This makes it somewhat similar to specimens in the *Desmopachriaubangoides* species group, but *Desmopachriadivergens* lacks the sexually dimorphic anterior clypeal margins of that group (males with the margin flattened and upturned, females beaded, but not as strongly modified (Miller 2001; Young 1980).

##### Description.

**Figures 1–12.**
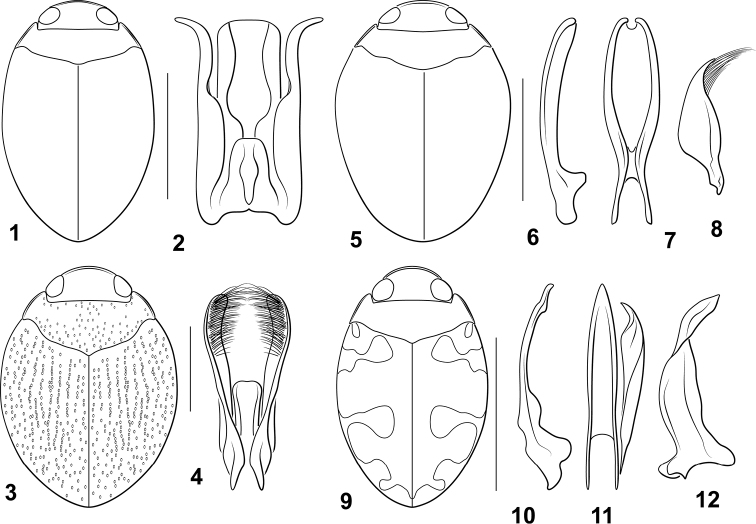
*Desmopachria* species. (**1, 2**) *Desmopachriadivergens***1** habitus **2** male genitalia, ventral aspect. (**3,4**) *Desmopachrialineata***1** habitus **2** male genitalia ventral aspect (**5–8**) *Desmopachriasurinamensis***5** habitus **6–8** male genitalia **6** median lobe, right lateral aspect **7** median lobe, ventral aspect **8** right lateral lobe, right lateral aspect (**9–12**) *Desmopachriatenua***9** habitus **10** median lobe, right lateral aspect **11** median lobe and right lateral lobe, ventral aspect; **12** right lateral lobe, right lateral aspect. Scale bars: 1.0 mm for habitus drawings.

***Measurements.***TL = 1.8–2.0 mm, GW = 1.1–1.2 mm, PW = 0.9–1.0 mm, HW = 0.6–0.7 mm, EW = 0.3–0.4 mm, TL/GW = 1.6–1.7, HW/EW = 2.1–2.2. Body elongate oval, laterally broadly curved, lateral margins somewhat discontinuous between pronotum and elytron, body broadest across elytra at midlength of body (Fig. 1).

***Coloration.*** Dorsal surface of head and pronotum evenly yellow-orange. Elytron evenly orange-brown, distinctly iridescent. Ventral surfaces orange, slightly darker medially on metathorax, medially slightly iridescent.

***Sculpture and structure.*** Head moderately broad, anteriorly rounded with anterior clypeal margin with narrow, continuous bead; surface of head shiny, finely and sparsely punctate; eyes large (HW/EW = 2.1–2.2); antennae short, scape and pedicel relatively large and rounded, flagellomere III long and slender, apically expanded, antennomeres IV-X short and broad, lobed at anterodorsal angle, antennomere XI elongate, apically pointed. Pronotum moderately short, lateral margins short, distinctly curved with continuous narrow bead, similar width throughout; surface shiny, very finely, evenly punctate. Elytron moderately broad, laterally broadly curved; surface shiny, more coarsely and evenly punctate than pronotum, punctation distinctive and prominent, densely and evenly punctate. Prosternum extremely short, longitudinally compressed, medially slightly carinate; prosternal process slender anteriorly, with distinctive, small medial tubercle, apically short and broad, medially flattened, apically broadly rounded. Metaventrite broad and evenly smoothly convex medially, surface shiny, finely, sparsely, and evenly punctate; metaventrite wings extremely slender. Metacoxa with medial portion short, < 1/3 length of metaventrite medially, metacoxal lines slightly divergent anteriorly; lateral portion of metacoxa extremely large, anteriorly strongly expanded; surface shiny, finely, sparsely, and evenly punctate. Metatrochanter large, subequal to length of ventral margin of metafemur; legs otherwise not noticeably modified. Abdomen with surfaces shiny and smooth, very finely and sparsely punctate.

***Male genitalia.*** Male median lobe in ventral aspect very broad, lateral margins linear to very broad, truncate apex (Fig. 2). Lateral lobe in ventral aspect slender, medially constricted on medial margin, apically slender, strongly curved laterad, apex narrowly rounded (Fig. 2).

***Sexual dimorphism.*** No obvious sexual dimorphic features were discovered.

***Variation.*** No characteristic variation was examined among the specimens examined.

##### Etymology.

This species is named *divergens*, Latin for divergent, for the apically distinctly divergent lateral lobes (Fig. 2).

##### Distribution.

Specimens are only known from the type locality, Caño Gato, Amazonas, Venezuela (Fig. 76).

##### Type material.

Holotype in MIZA, male labeled, “VENEZUELA: Amazonas State 4°58.838'N, 67°44.241'W, 95m Communidad Caño Gato, on Rio Sipapo; 16.i.2009; leg. Short, Miller, Camacho, Joly, & García VZ09-0116-01Z: along stream/ SM0843057 KUNHM-ENT [barcode label]/ HOLOTYPE *Desmopachriadivergens* Miller, 2021 [red label with black line border].” Paratypes, 19 in MIZA, MSBA, and SEMC labeled same as holotype except different barcode labels (Table 1) and each with “/…PARATYPE *Desmopachriadivergens* Miller, 2021 [blue label with black line border.”

**Table 1.** Barcode label data for *Desmopachria* species.

Species	Barcode Numbers
*Desmopachriaangulata*	SEMC0912629, SEMC0912722, SEMC0912731, SEMC0912736, SEMC0912739, SEMC0912744, SEMC0912748, SEMC0912750, SEMC0912765, SEMC0912779, SEMC0912790, SEMC0912877, SEMC0912904, SEMC0912907, SEMC0912908, SEMC0913073, SEMC0913151, SEMC0913157, SEMC0913158, SEMC0913179, SEMC0913184, SEMC0913191, SEMC0913199, SEMC0913353, SEMC0913357, SEMC0913361, SEMC0913363, SEMC0913367, SEMC0913375, SEMC0913377, SEMC0913387, SEMC0913400, SEMC0913430, SEMC0913438, SEMC0913500, SEMC0913501, SEMC0913507, SEMC0913516, SEMC0913517, SEMC0913519, SEMC0913521, SEMC0913522, SEMC0913526, SEMC0913527, SEMC0913530, SEMC0913534, SEMC0913539, SEMC0913541, SEMC0913542, SEMC0913546, SEMC0913550, SEMC0913551, SEMC0913552, SEMC0913553, SEMC0913556, SEMC0913560, SEMC0913561, SEMC0913562, SEMC0913563, SEMC0913568, SEMC0913610, SEMC0913652, SEMC0913657, SEMC0914252, SEMC0914256, SEMC0914259, SEMC0914281, SEMC0914285, SEMC0914287, SEMC0914301, SEMC0914302, SEMC0914305, SEMC0914309, SEMC0914310, SEMC0914317, SEMC0914320, SEMC0914325, SEMC0914340, SEMC0914344, SEMC0914349, SEMC0914359, SEMC0914360, SEMC0914362, SEMC0914390, SEMC0914399, SEMC0914594, SEMC0914606, SEMC0914619, SEMC0914640, SEMC0914648, SEMC0914660, SEMC0914677, SEMC0914749, SEMC0914789, SEMC0914803, SEMC0915003, SEMC0915004, SEMC0915030, SEMC0915448, SEMC0915591, SEMC0915603, SEMC0915767, SEMC0915769, SEMC0915773, SEMC0915781, SEMC0915952, SEMC0915954, SEMC0915957, SEMC0916019, SEMC0916192, SEMC0916426, SEMC0916947, SEMC1089075, SEMC1089077, SEMC1089078, SEMC1089088, SEMC1089091, SEMC1089167, SEMC1089298, SEMC1089353, SEMC1089364, SEMC1089367, SEMC1356172, SEMC1356175, SEMC1356181, SEMC1356182, SEMC1356187, SEMC1356194, SEMC1356206, SEMC1356428, SEMC1356497, SEMC1356956, SEMC1356958, SEMC1357114, SEMC1357402, SEMC1357404, SEMC1357405, SEMC1357406, SEMC1358166, SEMC1358167
*Desmopachriabisulcata*	SEMC0912025, SEMC0912028, SEMC0912165, SEMC0912168, SEMC0912177, SEMC0912178, SEMC0912190, SEMC0912194, SEMC0912195, SEMC0912196, SEMC0912199, SEMC0912327, SEMC0912340, SEMC0912341, SEMC0915067
*Desmopachriadivergens*	SM0842823, SM0842881, SM0843049, SM0843147, SM0843169, SM0843171, SM0843178, SM0843181, SM0843185, SM0843190, SM0843196, SM0843205, SM0843220, SM0843250, SM0843304, SM0843319, SM0843321, SM0843326, SM0843342
*Desmopachriaemarginata*	SEMC0908209, SEMC0908224, SEMC0908244, SEMC0915022, SEMC0930646, SEMC0930648, SEMC0930670, SEMC0930672, SEMC0930674, SEMC0930682, SEMC0930683, SEMC0930685, SEMC0930689, SEMC0930696, SEMC0930702, SEMC0930703, SEMC0930718, SEMC0930765, SEMC0934145, SEMC0934149, SEMC0965371, SEMC0965372, SEMC0965392, SEMC0965514, SEMC0965516, SEMC0965517, SEMC0965523, SEMC0965525, SEMC0965530, SEMC0965531, SEMC0965533, SEMC0965534, SEMC0965535, SEMC0965537, SEMC0965538, SEMC0965958, SEMC0965959, SEMC0965960, SEMC0966079, SEMC0966080, SEMC0966182, SEMC0966183, SEMC0966185, SEMC0966186, SEMC0966188, SEMC0966202, SEMC0966210, SEMC0966211, SEMC0966222, SEMC0966232, SEMC0966233, SEMC0966271, SEMC1046021, SEMC1046022, SEMC1046023, SEMC1080655, SEMC1080656, SEMC1080657, SEMC1080658, SEMC1080659, SEMC1080661, SEMC1080702, SEMC1080725, SEMC1080726, SEMC1080728, SEMC1080735, SEMC1080740, SEMC1080741, SEMC1080742, SEMC1080744, SEMC1080746, SEMC1080752, SEMC1080753, SEMC1080754, SEMC1080755, SEMC1080759, SEMC1080760, SEMC1080761, SEMC1080768, SEMC1080770, SEMC1080771, SEMC1080775, SEMC1080781, SEMC1080782, SEMC1080784, SEMC1114770, SEMC1114778, SEMC1114780, SEMC1114790, SEMC1114833, SEMC1114836, SEMC1114851, SEMC1234018, SEMC1234389, SEMC1234956, SEMC1234958, SEMC1234959, SEMC1234988, SEMC1236527, SEMC1236544, SEMC1315668, SEMC1315671, SEMC1315682, SEMC1315685, SEMC1315702, SEMC1315708, SEMC1315714, SEMC1315716, SEMC1315718, SEMC1328778, SEMC1328796, SEMC1328797, SEMC1328804, SEMC1328808, SEMC1328810, SEMC1328811, SEMC1328819, SEMC1328821, SEMC1328822, SEMC1328823, SEMC1328824, SEMC1328826, SEMC1328827, SEMC1328828, SEMC1328830, SEMC1328835, SEMC1328836, SEMC1328838, SEMC1328986, SEMC1328987, SEMC1328989, SEMC1328990, SEMC1328992, SEMC1328994, SEMC1328995, SEMC1328996, SEMC1328997, SEMC1328998, SEMC1328999, SEMC1329000, SEMC1329001, SEMC1329002, SEMC1329003, SEMC1329004, SEMC1329005, SEMC1329006, SEMC1329007, SEMC1329043, SEMC1329044, SEMC1357998, SEMC1357999, SEMC1358000, SM0827544
*Desmopachriaimpunctata*	SEMC1234012, SEMC1234013, SEMC1234014, SEMC1234015, SEMC1234016, SEMC1234017, SEMC1234019, SEMC1233985, SEMC1234021, SEMC1234022, SEMC1234011, SEMC1234037, SEMC1234008, SEMC1234009, SEMC1234005, SEMC1234006, SEMC1234004, SEMC1233952, SEMC1234003, SEMC1234002, SEMC1234001, SEMC1233966, SEMC1233947, SEMC1233948, SEMC1233950, SEMC1233951, SEMC1234034, SEMC1233960, SEMC1233965, SEMC1233958, SEMC1233964, SEMC1233969, SEMC1233970, SEMC1233971, SEMC1233972, SEMC1233973, SEMC1233975, SEMC1233976, SEMC1233977, SEMC1233989, SEMC1234035, SEMC1233968, SEMC1233981, SEMC1233980, SEMC1233979, SEMC1233978, SEMC1233983, SEMC1233974, SEMC1233986, SEMC1233987, SEMC1234036, SEMC1234000, SEMC1233999, SEMC1233993, SEMC1233994
	SEMC1233995, SEMC1233997, SEMC1233998, SEMC1233992, SEMC1233991, SEMC1233990, SEMC1233982, SEMC1114800, SEMC1114806, SEMC1114812, SEMC1080433, SEMC1080441, SEMC1080437, SEMC0930779, SEMC1080757, SEMC0930677, SEMC1080779, SEMC0965536, SEMC0965430, SEMC0966218, SEMC0966187, SEMC0966258, SEMC0966259, SEMC0966256, SEMC0966257, SEMC0966255, SEMC0966254, SEMC0966189, SEMC0966190, SEMC0966191, SEMC0966192, SEMC0966193, SEMC0966194, SEMC0966195, SEMC0966196, SEMC0966197, SEMC0966198, SEMC0966199, SEMC0966200, SEMC0966201, SEMC0966263, SEMC0966203, SEMC0966204, SEMC0966206, SEMC0966243, SEMC0966207, SEMC0966178, SEMC0966179, SEMC0966209, SEMC0966242, SEMC0966252, SEMC0966251, SEMC0966213, SEMC0966214, SEMC0966262, SEMC0966261, SEMC0966260, SEMC0966215, SEMC0966216, SEMC0966217, SEMC0966181, SEMC0966250, SEMC0966220, SEMC0966221, SEMC0966249, SEMC0966223, SEMC0966224, SEMC0966248, SEMC0966226, SEMC0966227, SEMC0966247, SEMC0966229, SEMC0966230, SEMC0966231, SEMC0966246, SEMC0966244, SEMC0966234, SEMC0966235, SEMC0966236, SEMC0966237, SEMC0966238, SEMC0966239, SEMC0966240, SEMC0966241, SEMC0966180, SEMC0966245, SEMC0966253, SEMC0966219, SEMC0965992, SEMC0965990, SEMC0965991, SEMC1234024, SEMC1234023, SEMC1234025, SEMC1234026, SEMC1234027, SEMC1234029, SEMC1234033, SEMC1113566, SEMC1113590, SEMC1113568, SEMC1113562, SEMC1113573, SEMC1113580, SEMC1113604, SEMC1114832, SEMC1113638, SEMC1113602, SEMC1114845, SEMC1113774, SEMC1113563, SEMC1113577, SEMC1113576, SEMC1114817, SEMC1114793, SEMC1114813, SEMC1113594, SEMC1113595, SEMC1113589, SEMC1113606, SEMC1114849, SM0843963, SEMC1233667, SEMC1233668, SEMC1233669, SEMC1233670, SEMC1233682, SEMC1233680, SEMC1233673, SEMC1233674, SEMC1233689, SEMC1233703, SEMC1233677, SEMC1233678, SEMC1233679, SEMC1233691, SEMC1233692, SEMC1233696, SEMC1233688, SEMC1233684, SEMC1233685, SEMC1233686, SEMC1233687, SEMC1233702, SEMC1233695, SEMC1233694, SEMC1080590, SEMC1080586, SEMC1080602, SEMC1080606, SEMC1080557, SEMC1080600, SEMC1080559, SEMC1080593, SEMC1080587, SEMC1080595, SEMC1080604, SEMC1080550, SEMC1080603, SEMC1080599, SEMC1080589, SEMC1233902, SEMC1233912, SEMC1233911, SEMC1233899, SEMC1233908, SEMC1233909, SEMC1233907, SEMC1233913, SEMC1233905, SEMC1080607, SEMC1233901, SEMC0966303, SEMC1114815, SEMC1114799
*Desmopachrialata*	USNMENT01190946, USNMENT01190947, USNMENT01190948, USNMENT01190949, USNMENT01190950, USNMENT01190951, USNMENT01190952, USNMENT01190953, USNMENT01190954, USNMENT01190955, USNMENT01190956, USNMENT01190959, USNMENT01190960, USNMENT01190961, USNMENT01190962, USNMENT01190963, USNMENT01190964, USNMENT01190965, USNMENT01190966, USNMENT01190967, USNMENT01190968
*Desmopachrialateralis*	SEMC0852875, SEMC0852886, SEMC0854130, SEMC0854136, SEMC0854137, SEMC0854145, SEMC0854146, SEMC0854147, SEMC0854148, SEMC0854149, SEMC0854154, SEMC0854155, SEMC0854156, SEMC0854173, SM0843335, USNMENT01187719, USNMENT01187720, USNMENT01187724, USNMENT01187725, USNMENT01187726, USNMENT01187727, USNMENT01187730, USNMENT01187731, USNMENT01187732, USNMENT01187733
*Desmopachriapseudocavia*	SEMC0854811, SEMC0854812, SEMC0854813, SEMC0891344, SEMC0891345, SEMC0891346, SEMC0891598, SEMC0891599, SEMC0891604, SEMC0893051, SEMC0907003, SEMC0907009, SEMC0907016, SEMC0907105, SM0831352, SM0831518, SM0831525, SM0842817, SM0842828, SM0842849, SM0842856, SM0842857, SM0842860, SM0842876, SM0842885, SM0842896, SM0842902, SM0842917, SM0842919, SM0842921, SM0842931, SM0842937, SM0842938, SM0842940, SM0842941, SM0842942, SM0842953, SM0842955, SM0842984, SM0842994, SM0842998, SM0843002, SM0843003, SM0843004, SM0843005, SM0843038, SM0843040, SM0843072, SM0843088, SM0843091, SM0843237, SM0843257, SM0843262, SM0843450, SM0843455, SM0843485, SM0843487, SM0843491, SM0843495, SM0843498, SM0843503, SM0843504, SM0843509, SM0843515, SM0843518, SM0843519, SM0843520, SM0843521, SM0843522, SM0843524, SM0843525, SM0843526, SM0843527, SM0843529, SM0843539, SM0843544, SM0843552, SM0843556, SM0843558, SM0843559, SM0843561, SM0843562, SM0843567, SM0843573, SM0843574, SM0845584, SM0845586, SM0845589, SM0845601, SM0845860, SM0845910, SM0846825, SM0846840
*Desmopachriasurinamensis*	SEMC1113335, SEMC1114816, SEMC1114818, SEMC1114835, SEMC1114841, SEMC1114843, SEMC1114844
*Desmopachriatruncata*	SEMC1087010, SEMC1087019, SEMC1087027, SEMC1087708, SEMC1087757, SEMC1087960, SEMC1087962, SEMC1087967, SEMC1087986, SEMC1088188, SEMC1088803, SEMC1088859, SEMC1088869, SEMC1088872, SEMC1089093, SEMC1328988, SEMC1328993
*Desmopachriawolfei*	SM0827524, SM0827525, SM0827526, SM0827528, SM0827531, SM0827533, SM0827536, SM0827538, SM0827540, SM0827678, SM0827679, SM0827682, SM0827687, SM0827690, SM0827691, SM0827693, SM0827697, SM0827699, SM0827712, SM0827719, SM0827723, SM0827731, SM0827732

#### ﻿ Desmopachria lineata sp. nov.

Taxon classificationAnimaliaColeopteraDytiscidae

3B7D6CEE-0A68-53B3-81D1-CCEA2DE9FE2A

http://zoobank.org/4B826E7E-1D9E-49C4-BEA3-42D3BA92962E

Figures 3 , 4 , 76

##### Type locality.

Venezuela, Amazonas State, near Iboruwa, “Tobogancito,” 5°48.414'N, 67°26.313'W.

##### Diagnosis.

This species is distinct in having coarse punctation on the pronotum and elytron with many punctures on the elytron arranged in distinctive longitudinal linear series, often confluent such that linear grooves are formed (Fig. 3). The male genitalia are distinctive with the median lobe shorter than half the length of the lateral lobes, broad and apically truncate and the lateral lobes elongate, apically somewhat expanded and with a dense series of elongate setae on the apicomedial surface (Fig. 4). Specimens are rather large for *Desmopachria* (TL = 2.6–2.8 mm). This species is similar to members of the *Desmopachriaportmanni* group, especially *Desmopachriagrammosticta* Braga & Ferreira‑Jr., which also has linear series of elytral punctures. However, *Desmopachrialineata* does not have the characteristic sexually dimorphic prosternal process of the *Desmopachriaportmanni* species-group (including *Desmopachriagrammosticta*). The prosternal process in males of *Desmopachrialineata* is not bifurcate with a medial pit, instead it is similar to the process in females. This is an unusual species in that it appears phonetically similar to members of the *Desmopachriaportmanni* group, but it lacks the bifid male prosternal process. It is certainly possible that the character states and relationships among these taxa is more complicated than currently understood. It seems clear that investigation of the utility and diversity of these and other characters among this group of *Desmopachria* should be investigated in the future.

##### Description.

***Measurements.***TL = 2.6–2.8 mm, GW = 1.9–2.0 mm, PW = 1.4–1.5 mm, HW = 1.0 mm, EW = 0.5–0.6 mm, TL/GW = 1.4, HW/EW = 1.8. Body large for genus, very broad, rounded, laterally broadly curved, lateral margins slightly continuous between pronotum and elytron, body broadest across elytra anterior at ca. midlength of body (Fig. 3).

***Coloration.*** Dorsal surface of head, pronotum and elytron red brown, moderately uniform in color throughout. Head appendages, pro- and mesothoracic legs and ventral surfaces of head and prothorax orange; other ventral surfaces red.

***Sculpture and structure.*** Head broad, anteriorly produced in rounded lobe; anterior margin of clypeus curved, flattened, margined with conspicuous, continuous flattened bead; surface of head shiny, finely but distinctly punctate over entire surface; eyes moderately large (HW/EW = 1.8); antennae short, scape and pedicel relatively large and rounded, flagellomere III long and slender, apically expanded, antennomeres IV-X short and broad, lobed at anterodorsal angle, antennomere XI elongate, apically pointed. Pronotum very short, lateral margins short, broadly curved, more so anteriorly, with continuous narrow bead; surface shiny, coarsely punctate medially and along most of anterior margin, less punctate lateromedially, punctures irregular, some confluent. Elytron moderately broad, laterally broadly curved; surface shiny, more coarsely punctate than pronotum, punctation distinctive and prominent, irregular, many punctures confluent forming distinctive longitudinal lines and grooves. Prosternum extremely short, longitudinally compressed, medially slightly carinate; prosternal process slender anteriorly, with distinctive, low medial tubercle, apical portion broad basally with broad basal U-shaped region and concave slender apical process emerging from between branches of U, apically narrowly rounded. Metaventrite broad and evenly convex medially, surface shiny, coarsely punctate, punctures forming longitudinal, linear series; metaventrite wings extremely slender. Metacoxa with medial portion short, < 1/3 length of metaventrite medially, metacoxal lines slightly divergent anteriorly; lateral portion of metacoxa extremely large, anteriorly strongly expanded; surface shiny, evenly and coarsely punctate. Metatrochanter large, subequal to length of ventral margin of metafemur; legs otherwise not noticeably modified. Abdomen with surfaces shiny and smooth, most of surface coarsely punctate.

***Male genitalia.*** Male median lobe in dorsal aspect short and broad, < 1/2 length of lateral lobe, apex slightly broadened, apically truncate with rounded lateral margins, medially very finely emarginate, (Fig. 4). Lateral lobe in dorsal aspect slender, elongate, evenly expanded apically and broadly curved mediad, apicomedial surface with dense, long setae (Fig. 4).

***Sexual dimorphism.*** No obvious sexual dimorphic features were discovered.

***Variation.*** There is some degree of variation in the punctation on the pronotum, elytron and ventral surfaces from specimen to specimen with some with punctures coarser, more confluent, with more strongly marked linear series. But in all cases, some degree of coarse linear series is present on the elytra and punctation is coarse overall compared with most other species in the genus.

##### Etymology.

This species is named *lineata*, Latin for lined, for the distinctive linear series of punctures on the disc of the elytron (Fig. 3).

##### Distribution.

This species is known from Amazonas and Bolivar States, Venezuela, and (Fig. 76).

##### Type material.

Holotype in MIZA, male labeled, “VENEZUELA: Amazonas State 5°48.414'N, 67°26.313'W, 80m nr. Iboruwa: “Tobogancito” 13.ii.2009; leg. K.B. Miller VZ09-0113-02E; leaf-choked detrital pools in forest/ SEMC0858759 KUNHM-ENT/ HOLOTYPE *Desmopachrialineata* Miller, 2021 [red label with black line border].” Paratypes, 2 in SEMC labeled, “VENEZUELA: Bolivar State 4°28.782'N, 61°34.904'W, 853m 1 km E. Pauji, trib. Of Rio Pauji 16.vii.2010;leg Short, Tellez, Arias along stream; VZ10-0716-01A/…” with barcode labels SEMC0906695 and SEMC0906722.

#### ﻿ Desmopachria surinamensis sp. nov.

Taxon classificationAnimaliaColeopteraDytiscidae

2127CC15-2895-5359-A7BB-07D117DCCA43

http://zoobank.org/1ECD49A5-390B-45B8-AA8E-1B1771B3DD23

Figures 6–8 , 76

##### Type locality.

Suriname, Sipaliwini District, Raleighvallen Nature Reserve, Voltzberg Trail, 4°40.910'N, 56°11.138'W.

##### Diagnosis.

This species is similar to members of the *Desmopachriaapicodente* group in having a longitudinal tumidity laterally on the elytron, being extremely broad (TL/GW = 1.3–1.4) and having a very distinctive, flattened bead along the anterior clypeal margin. However, *Desmopachriasurinamensis* lacks the apical tooth on the lateral lobe characteristic of the *Desmopachriaapicodente* group (see below). The male genitalia are distinctive. The median lobe in ventral aspect is elongate, broad, and comprised of long, slender, evenly curved lateral margins with a thin region in between (Fig. 7). The apices of the lateral struts are narrowly rounded and proximate with a small emargination in-between (Fig. 7).

##### Description.

***Measurements.***TL = 1.9–2.0 mm, GW = 1.4–1.5 mm, PW = 1.0–1.1 mm, HW = 0.6–0.7 mm, EW = 0.4–0.5 mm, TL/GW = 1.3–1.4, HW/EW = 1.8–1.9. Body very broad, rounded, laterally broadly curved, lateral margins continuous between pronotum and elytron, body broadest across elytra near midlength of body (Fig. 6).

***Coloration.*** Dorsal surface of head and pronotum orange. Elytron red, slightly paler anterolaterally. Head appendages, pro- and mesothoracic legs and ventral surfaces of head and prothorax orange, other ventral surfaces red.

***Sculpture and structure.*** Head broad, anteriorly produced, flattened, anterior margin of clypeus curved, margined with conspicuous, continuous flattened narrow bead; surface of head shiny, finely and sparsely punctate; eyes large (HW/EW = 1.8–19); antennae short, scape and pedicel relatively large and rounded, flagellomere III long and slender, apically expanded, antennomeres IV-X short and broad, lobed at anterodorsal angle, antennomere XI elongate, apically pointed. Pronotum very short, lateral margins short, slightly curved with continuous narrow bead, of even width throughout length; surface shiny, nearly impunctate medially, punctate around margins, punctation somewhat variable, with few larger punctures. Elytron moderately broad, laterally broadly curved; surface shiny, somewhat more coarsely and evenly punctate than pronotum, punctation fine, some punctures anteromedially on elytron forming moderately distinct longitudinal linear series; laterally with distinctive longitudinal rounded ridge extending posteriorly from humeral angle ~ 1/3 length of elytron. Prosternum extremely short, longitudinally compressed, medially slightly carinate; prosternal process slender anteriorly, with distinctive, small medial tubercle, apically short and broad, medially flattened, apically acutely pointed. Metaventrite broad and evenly smoothly convex medially, surface shiny, impunctate; metaventrite wings extremely slender. Metacoxa with medial portion short, ~ 1/3 length of metaventrite medially, metacoxal lines slightly divergent anteriorly; lateral portion of metacoxa extremely large, anteriorly strongly expanded; surface shiny, impunctate. Metatrochanter large, subequal to length of ventral margin of metafemur; legs otherwise not noticeably modified. Abdomen with surfaces shiny and smooth, very finely and sparsely punctate.

***Male genitalia.*** Male median lobe comprised of slender lateral margins between which is a membranous region, in lateral aspect entire median lobe broad, shallowly curved to rounded apex (Fig. 6); in ventral aspect, narrow basally, broad medially and apically, lateral margins converging, nearly touching apically, but separated by narrow, curved emargination (Fig. 7). Lateral lobe in lateral aspect short, broad, apically attenuated, and curved dorsad, with dense series of long, course setae along ventral margin to apex (Fig. 8).

***Sexual dimorphism.*** No clear sexual dimorphic features were discovered.

***Variation.*** No significant variation was examined among the specimens examined.

##### Etymology.

This species is named *surinamensis* after the country of collection of the type series.

##### Distribution.

This species is known only from Sipaliwini District, Suriname (Fig. 76).

##### Type material.

Holotype in NZCS, male labeled, “SURINAME: Sipaliwini District 04°40.910'N, 56°11.138'W, 78 m/ Raleighfallen [sic] Nature Reserve Voltzberg trail; margin of stream leg. C. Maier, V. Kadosoe 30.vii.2012; SR12-0730-01A/ SEMC1114775 KUNHM-ENT [barcode label]/ HOLOTYPE *Desmopachriasurinamensis* Miller, 2021 [red label with black line border].” Paratypes, 7, in SEMC and MSBA labeled same as holotype except with different SEMC barcode numbers (Table 1) and each with “/…PARATYPE *Desmopachriasurinamensis* Miller, 2021 [blue label with black line border.”

#### ﻿ Desmopachria tenua sp. nov.

Taxon classificationAnimaliaColeopteraDytiscidae

7F599838-F0CC-516B-818C-89232B620FAA

http://zoobank.org/EE94A378-D23A-420A-AE21-5C5D23E38846

Figures 9–12 , 76

##### Type locality.

Guyana, Region IX, Parabara, trail to mines, 2°05.095'N 59°14.174"W.

##### Diagnosis.

This species has a distinctive dorsal maculate color pattern (Fig. 9). Also, the male median lobe in lateral aspect is extremely slender (Fig. 10). The lateral lobes are moderately broad and twisted with the apical portion leaf-like (Figs 11, 12). The species does not fit into any of the recognized groups of *Desmopachria*, and it is not especially similar to any others in the “ungrouped” species.

##### Description.

***Measurements.***TL = 1.5 mm, GW = 1.0 mm, PW = 0.7 mm, HW = 0.5 mm, EW = 0.2 mm, TL/GW = 1.6, HW/EW = 2.1. Body oval, laterally broadly curved, lateral margins slightly discontinuous between pronotum and elytron, body broadest across elytra at ~ midlength of body (Fig. 9).

***Coloration*** (Fig. 9). Dorsal surface of head and pronotum pale orange; elytron orange-brown with pale orange maculae at humeral angle, anterobasally, subbasally near humeral angle, medially, along mediolateral margin, subapically along margin and at apex. Head appendages, pro- and mesothoracic legs and ventral surfaces of prothorax pale orange, maculae relatively well defined and variously confluent (Fig. 9); other ventral surfaces orange-brown.

***Sculpture and structure.*** Head broad, anteriorly produced, rounded; anterior margin of clypeus curved, flattened, margined with conspicuous, continuous narrow bead; surface of head shiny, very finely and sparsely punctate; eyes large (HW/EW = 2.1); antennae short, scape and pedicel relatively large and rounded, flagellomere III long and slender, apically expanded, antennomeres IV-X short and broad, lobed at anterodorsal angle, antennomere XI elongate, apically pointed. Pronotum short, lateral margins short, slightly curved with continuous narrow bead, slightly wider medially; surface shiny, finely, indistinctly punctate. Elytron moderately broad, laterally broadly curved; surface shiny, finely punctate throughout. Prosternum extremely short, longitudinally compressed, medially slightly carinate; prosternal process slender anteriorly, with distinctive, small medial tubercle, apically short and moderately broad, medially concave, apically pointed. Metaventrite broad and evenly smoothly convex medially, surface shiny, very finely punctate; metaventrite wings extremely slender. Metacoxa with medial portion short, < 1/3 length of metaventrite medially, metacoxal lines slightly divergent anteriorly; lateral portion of metacoxa extremely large, anteriorly strongly expanded; surface shiny, extremely finely punctate. Metatrochanter large, subequal to length of ventral margin of metafemur; legs otherwise not noticeably modified. Abdomen with surfaces shiny and smooth, very finely and sparsely punctate.

***Male genitalia.*** Male median lobe in lateral aspect long, extremely slender, and slightly curved, slightly expanded subapically on dorsal surface, apex narrowly pointed (Fig. 10); in ventral aspect slender, lateral margins evenly, broadly curved to pointed apex (Fig. 11). Lateral lobe in lateral aspect broad, apically twisted, apex leaflike and curved dorsad, apex pointed (Figs 11, 12).

***Sexual dimorphism and variation.*** Two specimens were examined, a male and one other. They are not noticeably different. It is not clear if the second specimen is a male or female.

##### Etymology.

This species is named *tenua*, Latin for slender, for the very thin male median lobe in lateral aspect.

##### Distribution.

This species is only known from the type locality in Guyana (Fig. 76).

##### Type material.

Holotype in CSBD, male labeled, “GUYANA: Region IX 2°05.095'N,59°14.174'W, 250m Parabara , Trail to mines detrital pools in forest leg. Short, Isaacs, Salisbury 2.xi.2013; GY13-1102-01A/ SEMC1271250 KUNHM-ENT [barcode label]/ HOLOTYPE *Desmopachriatenua* Miller, 2021 [red label with black line border].” Paratypes, 1 in SEMC labeled same as holotype except “…/ SEMC1271268 KINHM-ENT [barcode label]…” and “/…PARATYPE *Desmopachriatenua* Miller, 2021 [blue label with black line border.”

###### Checklist of ungrouped *Desmopachria* species

*Desmopachriaamrishi* Makhan, 2012 – Suriname

*Desmopachriaandreae* Megna & Sánchez-Fernández, 2014 – Cuba

*Desmopachriaattenuata* Régimbart, 1895 – Brazil (Braga and Ferreira-Jr. 2014)

*Desmopachriabalfourbrownei* Young, 1990 – Brazil (Braga and Ferreira-Jr. 2014)

*Desmopachriabarackobamai* Makhan, 2015 – French Guiana. Although described as being near *Desmopachriageijskesi*, this species appears more likely to be in the *Desmopachriaubangoides* species-group given the shape of the genitalia and the seemingly prominent male anterior clypeal margin in the illustrations provided (Makhan 2015: fig. 2). The description is inadequate to make a more definitive assessment, so the species is left ungrouped in *Desmopachria* (Miller 2001).

*Desmopachriadivergens* sp. nov. – Venezuela

*Desmopachriageijskesi* Young, 1990 – Suriname

*Desmopachriahylobates* Young, 1993 – Brazil

*Desmopachrianigrocapitata* Braga and Ferreira-Jr., 2010 – Brazil

*Desmopachrialineata* sp. nov. – Venezuela

*Desmopachriaparadoxa* Zimmermann, 1923 – Brazil

*Desmopachriarex* Gustafson & Miller, 2012 – Venezuela

*Desmopachriarishwani* Makhan, 2012 – Suriname

*Desmopachriasoesilae* Makhan, 2012 – Suriname

*Desmopachriastriga* Young, 1990 – Peru

*Desmopachriasubfasciata* Young, 1990 – Bolivia

*Desmopachriasurinamensis* sp. nov. – Suriname

*Desmopachriatambopatensis* Miller, 2005 – Peru

*Desmopachriataniae* Miller, 1999 – Bolivia

*Desmopachriatenua* sp. nov. – Guyana

### ﻿*Desmopachriaapicodente* species group

**Diagnosis.** The *Desmopachriaapicodente* group (a hereby newly identified group within *Desmopachria*) is characterized by the lateral lobe with a distinctive apical socketed spur or “tooth” that is directed apicomedially (Figs 17, 22, 27). Some species have a distinct lateral carina on the elytron dorsad to the epipleural carina (e.g., Figs 19, 24).

**Comments.** Another species, *Desmopachriasurinamensis* sp. nov., (see above) has a distinctive longitudinal lateral rounded tumidity, but this species lacks the apical tooth on the lateral lobe (Fig. 8). It is not known how widespread the longitudinal elytral tumidity character is in *Desmopachria*, but it is possible that further examination of this feature will be important for grouping of certain species in the genus. It should also be noted that the species, *Desmopachriaduodentata* Braga & Ferreira-Jr. (in the *Desmopachriaportmanni-portmanni* subgroup) also has two similar spurs apically on the lateral lobe.

#### ﻿ Desmopachria apicodente sp. nov.

Taxon classificationAnimaliaColeopteraDytiscidae

474981F0-5664-5E72-93DC-CC66BB5259DF

http://zoobank.org/55AFEE8C-DD1B-4E19-9B34-FB6DABA69C92

Figures 13–17 , 77

##### Type locality.

Venezuela, Apure State, between Orinoco and Cinaruco Rivers, 6°30.900'N, 67°32.604'W.

##### Diagnosis.

**Figures 13–27.**
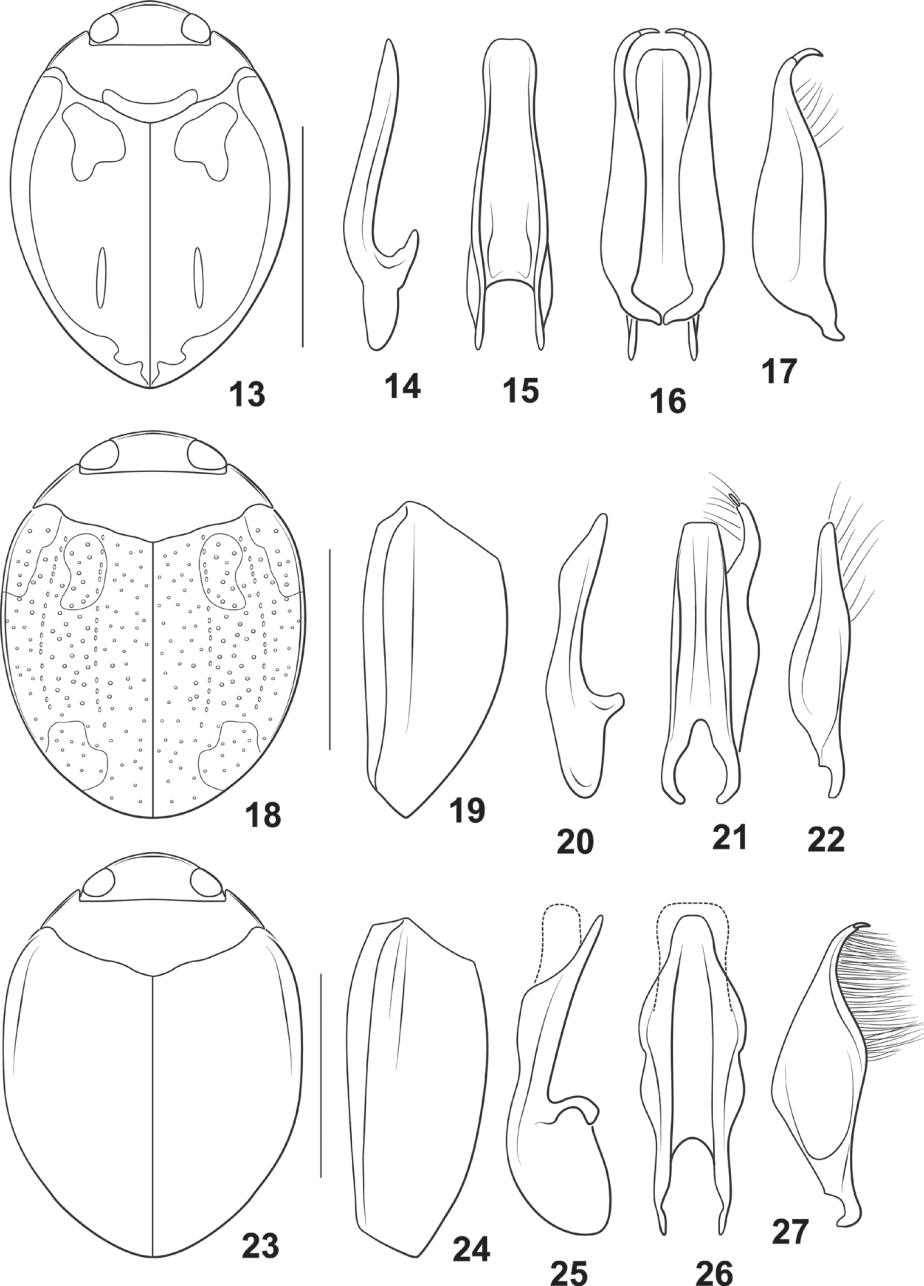
*Desmopachriaapicodente*-group species. (**13–17**) *Desmopachriaapicodente***13** habitus **14** male median lobe, right lateral aspect **15** male median lobe, ventral aspect **16** male median lobe and lateral lobes, ventral aspect **17** right lateral lobe, right lateral aspect (**18–22**) *Desmopachrialateralis***18** habitus **19** left elytron, left lateral aspect **20** male median lobe, right lateral aspect **21** male median lobe and right lateral lobe, ventral aspect **22** right lateral lobe, right lateral aspect (**23–27**) *Desmopachriatumida***23** habitus **24** left elytron, left lateral aspect **25** male median lobe, right lateral aspect **26** male median lobe, ventral aspect **27** right lateral lobe, right lateral aspect. Scale bars: 1.0 mm for habitus drawings.

Specimens are moderately large for a *Desmopachria* species (TL = 1.7–1.8 mm). The dorsal color pattern is distinctive with a large dark brown region on the elytron medially near the suture and with a large, diffuse pale macula apicomedially and along the lateral margins to the apex (Fig. 13). The male genitalia are distinctive with the male median lobe straight and apically sharply pointed in lateral aspect (Fig. 14) and broad and apically broadly truncate in ventral aspect (Figs 15, 16). The lateral lobe is moderately broad with the apices sharply curved mediad with a distinct, small, elongate apical tooth (Figs 16, 17). *Desmopachriaamyae* Miller, from Bolivia, has very similar genitalia including the apical tooth on the lateral lobe (Miller 2001: fig. 2), but that species has a different color pattern, an elytral sutural stria, and lateral furrows on the elytron (Miller 2001) which are missing in *Desmopachriaapicodente*. *Desmopachrialateralis*, also from Venezuela, also has a minute apical tooth on the lateral lobe, but a different dorsal color pattern, a distinct carina along the lateral margin of the elytron, and the male median lobe is differently shaped, with a distinctive subapical expansion on the ventral margin medially (Fig. 20).

##### Description.

***Measurements.***TL = 1.7–1.8 mm, GW = 1.2–1.3 mm, PW = 1.0–1.1 mm, HW = 0.7–0.8 mm, EW = 0.4–0.5 mm, TL/GW = 1.3–1.4, HW/EW = 1.8–1.9. Body broad, laterally broadly rounded, lateral margins continuous between pronotum and elytron; dorsoventrally rounded.

***Coloration.*** Head pale orange. Pronotum pale orange with dark area along posterior margin. Elytron dark red-brown broadly along suture, becoming pale red medially, with pale, poorly demarked maculae anteromedially, laterally along border, apically, and often in small line medially (Fig. 13). Ventral surfaces yellow to orange.

***Sculpture and structure.*** Head broad, short; anterior margin of clypeus finely margined with continuous narrow bead; surface of head shiny, punctation extremely fine and sparse; eyes large (HW/EW = 1.8–1.9); antennae short, scape and pedicel relatively large and rounded, flagellomere III long and slender, apically expanded, antennomeres IV-X short and broad, antennomere XI elongate, apically pointed. Pronotum short, lateral margins short, curved with continuous narrow bead; surface shiny, punctation very fine, of the same size and evenly distributed, posterior margin slightly sinuate. Elytron broad, laterally broadly curved; surface shiny, punctation small, some punctures arranged into indistinct series, especially anteromedially. Prosternum extremely short, longitudinally compressed, medially flattened; prosternal process short, broad, flattened, apically pointed. Metaventrite broad and evenly smoothly convex medially, surface shiny, impunctate; metaventrite wings extremely slender. Metacoxa with medial portion short, < 1/3 length of metaventrite medially, metacoxal lines slightly sinuate, divergent anteriorly; lateral portion of metacoxa extremely large, anteriorly strongly expanded; surface shiny, impunctate. Metatrochanter very large, longer than length of ventral margin of metafemur; legs otherwise not noticeably modified. Abdomen with surfaces shiny and smooth, surface impunctate.

***Male genitalia.*** Male median lobe in lateral aspect moderately broad, elongate, straight, and of even width to sharply pointed apex, apically slight curved ventrad (Fig. 14; in ventral aspect elongate and broad, subapically slightly constricted, apex broadly subtruncate (Fig. 15). Lateral lobe elongate, moderately broad throughout most of length, apex strongly curved mediad with small but distinctive elongate “tooth” at apex (Figs 16, 17).

***Sexual dimorphism.*** No obvious external sexual dimorphism was observed.

***Variation.*** Specimens vary considerably in the extent of the fasciate pattern on the elytron and intensity of coloration, some of which is related to teneral condition. Some specimens barely have pale regions visible that are weakly delimited, others have a distinctive pattern on the dorsal surface.

##### Etymology.

This species is named *apicodente*, from Latin, *apico*, for apical, and *dente*, for tooth, for the apical tooth on the male lateral lobe.

##### Distribution.

This species is known only from Apure, Bolivar and Amazonas States, Venezuela and Region IX, Guyana (Fig. 77).

##### Type material.

Holotype in MIZA, male labeled, “VENEZUELA: Apure State 6°30.900'N, 67°32.604'W; 68m Btw Orinoco and Cinaruco Rivers 17.i.2009; Short, Miller, Camacho VZ09-0117-01X; morichal/ SMEC085232 KUNHM-ENT [barcode label]/ HOLOTYPE *Desmopachriaapicodente* Miller, 2021 [red label with black line border].” Paratypes, 400, all with different barcode labels (Table 1) and “…/PARATYPE *Desmopachriaapicodente* Miller, 2021 [blue label with black line border]”; 20 in MIZA, MSBA and SEMC labeled same as holotype; 31 in SEMC labeled, “VENEZUELA: Bolívar State 6°13'4.6"N, 67°14'26.4"W, 60m ca. 25 km E El Burro 12.i.2009; leg. Short et al. rocky morichal; VZ09-0113-01X/…”; 10 in SEMC labeled, “VENEZUELA: Amazonas State 5°20.514'N; 67°45.315'W, 87m S. Communidad Porvenir 15.i.2009; leg. Miller & Short VZ09-0115-03B: small streamlet/…”; 50 in CSBD and SEMC labeled, “GUYANA: Region IX 2°48.531'N,59°51.900'W, 170m Kusad Mts., Mokoro Creek pool in rock, with detritus leg. A. Short; 27.x.2013 GY13-1027-03E/…”; 29 in SEMC labeled, “GUYANA: Region IX 2°47.417'N, 59°53.986'W, 113m Kusad Mts., Taraara Wao Creek margin & isolated side pools leg. Short, Isaacs, Salisbury 28.x.2013; GY13-1028-01A/…”; 9 in SEMC labeled, “GUYANA: Region IX 2°48.588'N,59°51.931'W, 194m Kusad Mts., basecamp area leg. A. Short; 23.x.2013; pool in creek bed; GY13-1023-02A/…”; 7 in SEMC labeled, “GUYANA: Region IX 2°48.531'N,59°51.900'W, 170m Kusad Mts., Mokoro Creek pool in rock, with detritus leg. A. Short; 27.x.2013 GY13-1027-03E/…”; 12 in SEMC labeled same, except, “…main seepage area leg. Short, Isaacs, Salisbury 27.x.2013; GY13-1027-03B/…”; 1 in SEMC labeled, “GUYANA: Region IX 2°48.531'N,59°51.900'W, 170m Kusad Mts., large seepage nr. Basecamp; on wet rocks leg. A. Short & W. Washington GY13-1024-03C/…”

#### ﻿ Desmopachria lateralis sp. nov.

Taxon classificationAnimaliaColeopteraDytiscidae

5D470318-FC9E-585C-B80A-CED409A93908

http://zoobank.org/C6485BE5-4B33-45C6-94E2-F16B26364EA9

Figures 18–22 , 77

##### Type locality.

Venezuela, Amazonas State, Communidad Caño Gato, on Rio Sipapo, 4°58.838'N, 67°44.341'W.

##### Diagnosis.

This species is particularly distinctive because of the lateral carina extending along the lateral margin of the elytron from the humeral angle almost the entire length of the elytron. Specimens are moderately large for *Desmopachria* species (TL = 1.7–1.8 mm). The dorsal color pattern is distinctive in many specimens with most of the elytron brown with a broad region longitudinally along the suture dark brown (Figs 18, 19). The male genitalia are distinctive with the median lobe in lateral aspect elongate, sublinear along the dorsal margin and medially expanded along the ventral margin and apically narrowly rounded (Fig. 20). In ventral aspect the median lobe is broad with the lateral margins slightly convergent to the broadly truncate apex (Fig. 21). The lateral lobe is moderately broad basally and narrowed apically to a narrowly rounded apex, apically with a small, elongate apical tooth (Fig. 22). See under *Desmopachriaapicodente* for additional diagnostic differences between that species, *Desmopachriaamyae*, and *Desmopachrialateralis*.

##### Description.

***Measurements.***TL = 1.7–1.8 mm, GW = 1.3–1.4 mm, PW = 1.0–1.1 mm, HW = 0.6–0.7 mm, EW = 0.3–0.4 mm, TL/GW = 1.3–1.4, HW/EW = 1.9–2.0. Body very broad, rounded, laterally broadly rounded, lateral margins slightly discontinuous between pronotum and elytron; dorsoventrally somewhat compressed.

***Coloration.*** Head pale orange. Pronotum pale orange with narrow dark area medially along posterior margin. Elytron dark orange, with broad dark brown band longitudinally along suture, also with small diffuse, pale macula at apex, and paler diffuse areas anteromedially (Fig. 18). Ventral surfaces yellow to orange.

***Sculpture and structure.*** Head broad, short; anterior margin of clypeus finely margined with continuous narrow bead, bead slightly more expanded medially; surface of head shiny, punctures extremely fine, nearly impunctate; eyes large (HW/EW = 1.9–2.0); antennae short, scape and pedicel relatively large and rounded, flagellomere III long and slender, apically expanded, antennomeres IV-X short and broad, antennomere XI elongate, apically pointed. Pronotum short, lateral margins short, curved with continuous narrow bead; surface shiny, punctation very fine over most of surface, larger and denser posteromedially. Elytron broad, laterally broadly curved; surface shiny, punctation variable, some punctures arranged into indistinct series, especially anteromedially (Fig. 18); lateral margin, dorsad of epipleural carina, with distinct, longitudinal carina extending from humeral angle most of length of elytron (Fig. 19). Prosternum extremely short, longitudinally compressed, medially flattened; prosternal process short, broad, flattened, apically pointed. Metaventrite broad and evenly smoothly convex medially, surface shiny, impunctate; metaventrite wings extremely slender. Metacoxa with medial portion short, < 1/3 length of metaventrite medially, metacoxal lines slightly sinuate, divergent anteriorly; lateral portion of metacoxa extremely large, anteriorly strongly expanded; surface shiny, impunctate, slightly rugulose. Metatrochanter very large, longer than length of ventral margin of metafemur; legs otherwise not noticeably modified. Abdomen with surfaces shiny and smooth, surface impunctate.

***Male genitalia.*** Male median lobe in lateral aspect moderately broad, elongate, straight, with submedial expansion on ventral surface, apex narrowly rounded (Fig. 20); in ventral aspect elongate and broad, apex broadly subtruncate, slightly emarginate (Fig. 21). Lateral lobe elongate, moderately broad medially, apically narrowed to apex, apex sharply curved mediad with small but distinctive elongate “tooth” at apex (Fig. 22).

***Sexual dimorphism.*** No obvious external sexual dimorphism was observed.

***Variation.*** Specimens vary in the extent of the color pattern on the elytron and intensity of coloration, some of which is related to teneral condition. Some specimens have pale regions barely visible and they are weakly delimited, others have a distinctive pattern on the dorsal surface.

##### Etymology.

This species is named *lateralis*, from the Latin for the lateral carina on the elytron.

##### Distribution.

This species is known only from Amazonas State, Venezuela (Fig. 77).

##### Type material.

Holotype in MIZA, male labeled, “VENEZUELA: Amazonas State 4°58.838'N, 67°44.341'W; 95m Communidad Caño Gato, on Rio Sipapo; 16.i.2009; leg. Short, Miller, Camacho, Joly, & García VZ09-0116-01X; along stream/ SM0843192 KUNHM-ENT [barcode label]/ HOLOTYPE *Desmopachrialateralis* Miller, 2021 [red label with black line border].” Paratypes, 1 in SEMC labeled same as holotype except with “…/ SM0843335 KUNHM-ENT [barcode label];” 14 in MSBA, MIZA, and SEMC labeled, “VENEZUELA: Amazonas State 5°20.514'N; 67°45.315'W, 87m S. Communidad Porvenir 15.i.2009; leg. Miller & Short VZ09-0115-03B; small streamlet…”; 10 in USNM and MSBA labeled, “BRAZIL: Para:Rio Zingu Camp (52°22'W. 3°39'S) Altamira (ca 60km S.) 11 Oct 2986 P.Spangler & R.Crombie/…” Each paratype with different barcode labels (Table 1) and all paratypes with “…PARATYPE *Desmopachrialateralis* Miller, 2021 [blue label with black line border].”

#### ﻿ Desmopachria tumida sp. nov.

Taxon classificationAnimaliaColeopteraDytiscidae

4D7DDCB5-7871-55D1-B49B-C0702346EF47

http://zoobank.org/984AAC32-D689-4CD8-BD12-382AFEF9DB5E

Figures 23–27 , 77

##### Type locality.

Venezuela, Bolivar State, Gran Sabana, Pauji, Esmeraldes, 4°28.233'N, 17°35.559'W.

##### Diagnosis.

This species is characterized by the distinctive lateral longitudinal tumidity on the elytron (Figs 23, 24). The humeral angle in dorsal aspect is subsinuate because of lateral expansion of the tumidity. The male genitalia are distinct with the median lobe broad with the lateral margins medially bisinuate and apically narrowed to an apically rounded apex (Fig. 25) and in lateral aspect with the base large and rounded and the apical portion with the dorsal margin linear and with a broad expansion on the ventral margin with the apex slender and apically narrowly rounded (Fig. 26). The lateral lobe is broad medially and apically tapered to a pointed apex with a distinct apical socketed “tooth” (Fig. 27). This tooth is shared with two other species, *Desmopachriaapicodente* sp. nov. and *Desmopachrialateralis* sp. nov., together making up the *Desmopachriaapicodente* species-group (see Diagnosis under *Desmopachriaapicodente* for discussion of differences). *Desmopachrialateralis* also has a prominent lateral longitudinal elytral tumidity, but that species has differently shaped male genitalia (Figs 20–22).

##### Description.

***Measurements.***TL = 2.0 mm, GW = 1.5 mm, PW = 1.1 mm, HW = 0.7 mm, EW = 0.4 mm, TL/GW = 1.4, HW/EW = 1.8. Body very broad, laterally broadly rounded, lateral margins nearly continuously curved between pronotum and elytron (Fig. 23).

***Coloration.*** Head and pronotum evenly orange. Elytron brown, laterally and apically somewhat paler brown-orange. Ventral surfaces and appendages orange to orange-brown.

***Sculpture and structure.*** Head broad, short; anterior margin of clypeus distinctly margined with continuous flattened bead, broader and flatter medially; surface of head shiny, punctation extremely fine and sparse; eyes large (HW/EW = 1.8); antennae short, scape and pedicel relatively large and rounded, flagellomere III long and slender, apically expanded, antennomeres IV-X short and broad, antennomere XI elongate, apically pointed. Pronotum short, lateral margins short, gently curved with continuous narrow marginal bead; surface shiny, punctation very fine, of the same size and evenly distributed except more coarsely punctate posteromedially. Elytron broad, laterally broadly curved, lateral margin distinctly sinuate at humeral angle, with distinct lateral tumidity extending posteriorly from humeral angle (Figs 23, 24); surface shiny, punctation of two sizes, minute and small, evenly punctate. Prosternum extremely short, longitudinally compressed, medially flattened; prosternal process broad, flattened, concave with broad lateral bead, apically pointed. Metaventrite broad and evenly smoothly convex medially, surface shiny, impunctate; metaventrite wings extremely slender. Metacoxa with medial portion short, < 1/3 length of metaventrite medially, metacoxal lines slightly sinuate, divergent anteriorly; lateral portion of metacoxa extremely large, anteriorly strongly expanded; surface shiny, extremely minutely punctate. Metatrochanter very large, longer than length of ventral margin of metafemur; legs otherwise not noticeably modified. Abdomen with surfaces shiny and smooth, surface impunctate.

***Male genitalia.*** Male median lobe in in lateral aspect with large, broad, rounded base, apical portion with dorsal margin linear, ventral margin with large expansion medially, apically narrowed and slender to narrowly rounded apex (Fig. 25); in ventral aspect broad, long, lateral margins distinctly sinuate medially, apically narrowed to broadly rounded apex (Fig. 26). Lateral lobe large, broad medially, narrow basally and apically strongly and evenly narrowed to pointed apex, dorsal margin with long dense series of long setae (Fig. 27).

***Sexual dimorphism and variation.*** Only a single male specimen was examined.

##### Etymology.

This species is named *tumida*, Latin for swollen, for the laterally tumid, or swollen, elytral margins (Figs 23, 24).

##### Distribution.

This species is known only from one site in the Gran Sabana of Bolivar State, Venezuela (Fig. 77).

##### Type material.

Holotype in MIZA, male labeled, “VENEZUELA: Bolivar State 4°28.233'N, 61°35.559'W, 867 m Gran Sabana, Pauji: Esmeraldes 16.vii.2010;leg. Short, Tellez, Arias detrital pools by forested stream VZ10-0716-02A/ SEMC0908227 KUNHM-ENT [barcode label]/ HOLOTYPE *Desmopachriatumida* Miller, 2021 [red label with black line border].” This species is only known from the holotype.

###### Checklist of *Desmopachriaapicodente* species group

*Desmopachriaapicodente* sp. nov. – Guyana, Venezuela

*Desmopachrialateralis* sp. nov. – Venezuela

*Desmopachriatumida* sp. nov. – Venezuela

### ﻿*Desmopachriabifurcita* species group

**Diagnosis.** The *Desmopachriabifurcita* group (a newly identified group within *Desmopachria*) is characterized by the median lobe very short and stout and the lateral lobes long, broad, flattened, and medially bent dorsad (e.g., Figs 29, 30). Some species have the basal portion of the lateral lobe broad, and others smaller, but the apical half in these species in lateral aspect is linear and slender to the apex (e.g., Fig. 30).

**Comments.** This new species group, derived from two new species and several species previously not placed in a defined species group in the genus, is diagnosed by similar male genitalia (see above). The group does not have many other similarities. Some are dorsally maculate, others are not. They are of somewhat variable shape. More investigation will be needed to determine the naturalness of this grouping. In addition, other species of *Desmopachria* also have similar genitalia. *Desmopachriachei* Miller has diagnostically similar genitalia (Miller 1999: fig. 1) but has a subsutural stria, so is placed in the *Desmopachriastriola* species group (Miller 1999). *Desmopachriamendozana* (Steinheil) also has similar genitalia (Young 1980: figs 2, 3) but has the anterior metatibial spur serrate, so is placed in the *Desmopachriavicina* species group (Miller 2001; Young 1980).

#### ﻿ Desmopachria bifurcita sp. nov.

Taxon classificationAnimaliaColeopteraDytiscidae

5AE03040-0D3A-529C-A54F-EBA78AC635D7

http://zoobank.org/B3351A38-F6E8-41FB-BCAA-DC78CB00A8F8

Figures 28–30 , 78

##### Type locality.

Peru, Junín, Sani Beni. The type locality is ambiguous. According to Otto (2017) this collection locality by P. Woytkowski may refer to a misspelling of “Sani Benu” which is at approximately 11.253917°S, 74.565565°W (see below).

##### Diagnosis.

Males of this species have the male genitalia very distinctive with the median lobe very short and strongly bifurcate (Fig. 29). The lateral lobes in dorsal aspect and broad, parallel-sided, and apically subtruncate (Fig. 29). In lateral aspect, they are slender and abruptly bent medially (Fig. 30). Specimens are very broad in dorsal aspect and dark reddish-brown dorsally. A number of species of *Desmopachria* have the median lobe short with long, medially bent lateral lobes like *Desmopachriabifurcita*, including *Desmopachriabifasciata* Zimmermann, *Desmopachriabolivari* Miller, *Desmopachriachei* Miller, *Desmopachrialata* sp. nov., *Desmopachriavarians* Wehncke, and *Desmopachriaovalis* Sharp. Among these, *Desmopachriabifurcita* is the only one with a bifid median lobe (Fig. 29). These species may together form another species group within *Desmopachria*, but more thorough examination of specimens will be needed to determine this.

##### Description.

**Figures 28–45.**
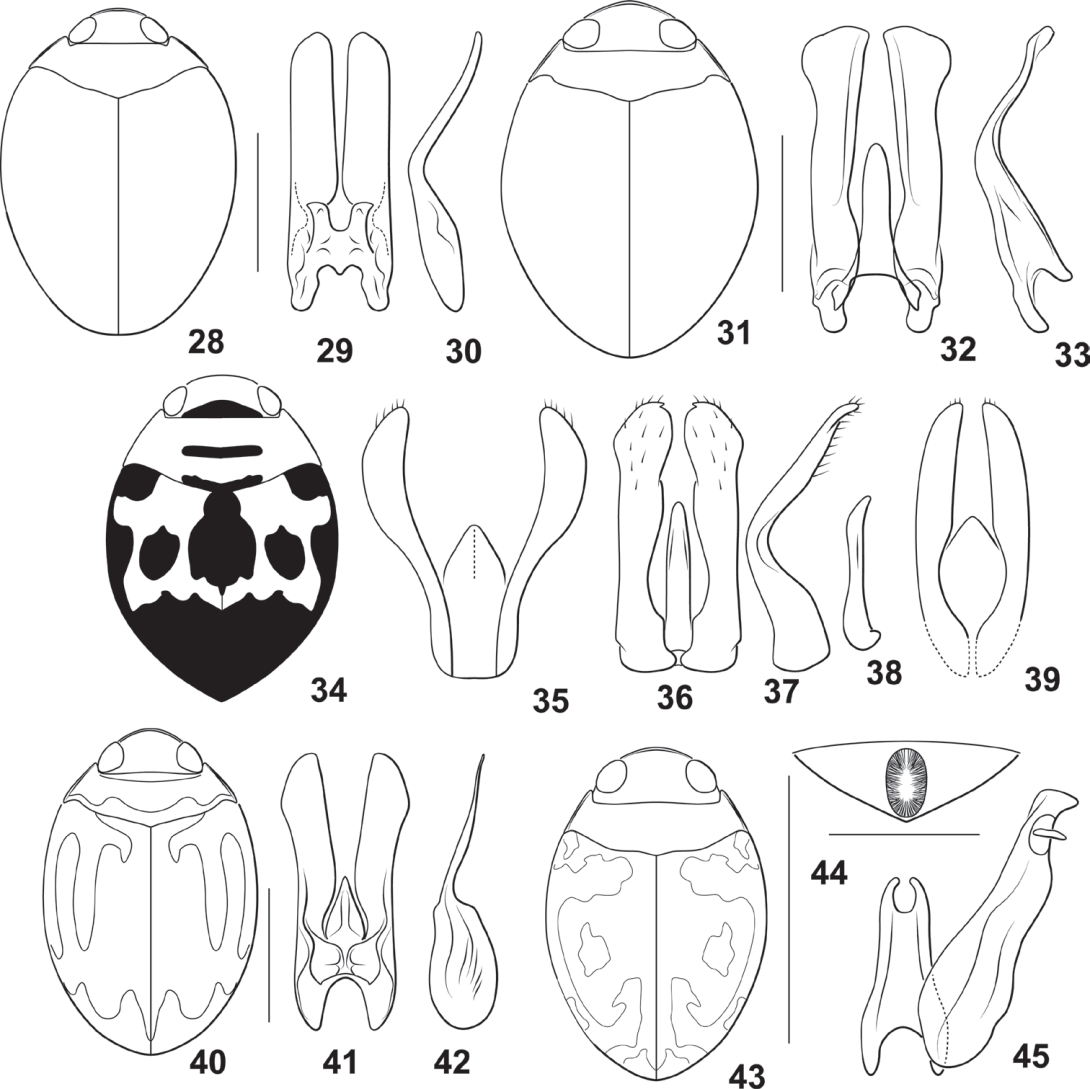
*Desmopachria* species. (**28–30**) *Desmopachriabifurcita***28** habitus **29** male median and lateral lobes, dorsal aspect **30** male lateral lobe, right lateral aspect (**31–33**) *Desmopachrialata***31** habitus **32** male genitalia, ventral aspect **33** right lateral lobe, right lateral aspect. (**34, 35**) *Desompachriabifasciata***34** habitus **35** male genitalia, ventral aspect (**36–38**) *Desmopachriabolivari***36** male genitalia, dorsal aspect **37** right lateral lobe, right lateral aspect **38** median lobe, right lateral aspect **39***Desmopachriaovalis*, male genitalia, dorsal aspect (**40–42**) *Desmopachriavarians***40** habitus **41** male genitalia, ventral aspect **42** right lateral lobe, right lateral aspect (**43–45**) *Desmopachriapseudocavia***43** habitus **44** abdominal sternite VI, ventral aspect **45** male median lobe and right lateral lobe, ventral aspect. Scale bars: 1.0 mm for habitus drawings; 0.25 mm (**44**).

***Measurements.***TL = 1.8–1.9 mm, GW = 1.3 mm, PW = 1.0 mm, HW = 0.6 mm, EW = 0.3 mm, TL/GW = 1.4, HW/EW = 2.1–2.4. Body very broad, rounded, laterally broadly curved, lateral margins continuous between pronotum and elytron, body broadest across elytra anterior midlength of body (Fig. 28).

***Coloration*** (Fig. 28). Dorsal surface of head dark red, paler red along anterior margin. Pronotum evenly red. Elytron red, vaguely darker along anterior and sutural margins. Head appendages, legs, and ventral surfaces red to dark red.

***Sculpture and structure.*** Head broad, anteriorly produced in rounded lobe; anterior margin of clypeus curved, flattened, margined with conspicuous, continuous flattened narrow bead; surface of head shiny, finely and sparsely punctate; eyes large (Fig. 28, HW/EW = 2.1–2.4); antennae short, scape and pedicel relatively large and rounded, flagellomere III long and slender, apically expanded, antennomeres IV-X short and broad, lobed at anterodorsal angle, antennomere XI elongate, apically pointed. Pronotum very short, lateral margins short, slightly curved with continuous narrow bead, slightly wider medially; surface shiny, impunctate medially, punctate around margins, punctation variable, fine to course. Elytron moderately broad, laterally broadly curved; surface shiny, more coarsely and evenly punctate than pronotum, punctation distinctive and prominent. Prosternum extremely short, longitudinally compressed, medially slightly carinate; prosternal process slender anteriorly, with distinctive, small medial tubercle, apically short and broad, medially slightly carinate, concave, apically acutely pointed. Metaventrite broad and evenly smoothly convex medially, surface shiny, moderately and irregularly punctate; metaventrite wings extremely slender. Metacoxa with medial portion short, < 1/3 length of metaventrite medially, metacoxal lines slightly divergent anteriorly; lateral portion of metacoxa extremely large, anteriorly strongly expanded; surface shiny, evenly punctate, punctures evenly distributed. Metatrochanter large, subequal to length of ventral margin of metafemur; legs otherwise not noticeably modified. Abdomen with surfaces shiny and smooth, very finely and sparsely punctate.

***Male genitalia.*** Male median lobe in lateral aspect extremely short, apically distinctly bifid, each branch apicolaterally pointed, (Fig. 29). Lateral lobe in ventral aspect evenly broad throughout length, lateral margins parallel, apically broadly rounded (Fig. 30) in lateral aspect slender throughout length, abruptly curved medially (Fig. 31).

***Sexual dimorphism.*** No obvious sexual dimorphic features were discovered.

***Variation.*** No characteristic variation was examined among the specimens examined.

##### Etymology.

This species is named *bifurcita*, Latin for the short, bifurcate male median lobe.

##### Distribution.

The exact locality of collection of this species is somewhat ambiguous. The locality Sani Beni is probably “Sani Benu” at coordinates 11.253917°S, 74.565565°W, as with Eucnemidae Eschscholtz specimens collected by the same collector as documented by Otto (2017) (Fig. 78).

##### Type material.

Holotype in SEMC, male labeled, “PERU Dept. Juni Sani Beni 1 Aug. 1935 [“1 Aug.” handwritten] P. Wyotkowski co/ HOLOTYPE *Desmopachriabifurcita* Miller, 2021 [red label with black line border].” Paratypes, 5 in SEMC and MSBA labeled same as holotype except with dates, 10 Aug 1935, 11 Aug 1935, 19 Aug 1935, 20 Aug 1935, and 6 Nov 1935 and “/…PARATYPE *Desmopachriabifurcita* Miller, 2021 [blue label with black line border].”

#### ﻿ Desmopachria lata sp. nov.

Taxon classificationAnimaliaColeopteraDytiscidae

1F65DD47-08BE-58E3-BBBE-DCEFD8F011A8

http://zoobank.org/3D15426D-6C1A-461D-A567-B0A1F59E31D7

Figures 31–33 , 78

##### Type locality.

Brazil, Pará State, Cachimbo.

##### Diagnosis.

Males of this species have the median lobe short and simple (Fig. 32). The lateral lobes are broad, long, and apically broadly expanded and truncate in dorsal aspect (Fig. 32) and medially abruptly bent and apically sinuate with a narrowly rounded apex (Fig. 33). The genitalia are similar to *Desmopachriabolivari* Miller, but that species has the apices of the lateral lobes broadened and with a distinctive medially directed tooth or hook, among other more subtle genitalic shape differences (Miller 1999: fig. 4).

##### Description.

***Measurements.***TL = 2.3 mm, GW = 1.5–1.6 mm, PW = 1.2–1.3 mm, HW = 0.8 mm, EW = 0.4 mm, TL/GW = 1.4–1.5, HW/EW = 2.1–2.2. Body very broad, rounded, laterally broadly curved, lateral margins continuous between pronotum and elytron, body broadest across elytra near midlength of body (Fig. 31).

***Coloration*** (Fig. 31). Dorsal surface of head, pronotum and elytron dark red-brown, slightly but distinctly paler laterally on pronotum. Head appendages, pro- and mesothoracic legs, and prothoracic ventral surfaces red-orange, other ventral surfaces and metathoracic legs dark red-brown.

***Sculpture and structure.*** Head broad, anterior margin flattened, margined with distinctive narrow bead; surface of head shiny, finely and sparsely punctate; eyes large (Fig. 31, HW/EW = 2.1–2.2); antennae short, scape and pedicel relatively large and rounded, flagellomere III long and slender, apically expanded, antennomeres IV-X short and broad, lobed at anterodorsal angle, antennomere XI elongate, apically pointed. Pronotum very short, lateral margins short, slightly curved with continuous narrow bead, slightly wider medially; surface shiny, impunctate medially, finely punctate around margins. Elytron very broad, laterally broadly curved; surface shiny, more coarsely and evenly punctate than pronotum, punctation distinctive and prominent, moderately fine. Prosternum extremely short, longitudinally compressed, medially slightly carinate; prosternal process slender anteriorly, with distinctive, small medial tubercle, apically short and moderately broad, medially evenly convex, apically broadly pointed. Metaventrite broad and evenly smoothly convex medially, surface shiny, impunctate; metaventrite wings extremely slender. Metacoxa with medial portion short, < 1/3 length of metaventrite medially, metacoxal lines indistinct, slightly divergent anteriorly; lateral portion of metacoxa extremely large, anteriorly strongly expanded; surface shiny, evenly, finely punctate. Metatrochanter large, subequal to length of ventral margin of metafemur; legs otherwise not noticeably modified. Abdomen with surfaces shiny and smooth, very finely and sparsely punctate.

***Male genitalia.*** Male median lobe in lateral aspect short, slightly curved, apically simple; in ventral aspect short, slender, parallel-sided to narrowly rounded apex (Fig. 32). Lateral lobe in ventral aspect evenly broad throughout much of length, apex broadly expanded laterally, apex broad, somewhat obliquely truncate (Fig. 32); in lateral aspect slender throughout length, abruptly curved medially, apex abruptly slightly expanded and apically narrowly rounded (Fig. 33).

***Sexual dimorphism.*** No obvious sexual dimorphic features were discovered.

***Variation.*** There is some variation in the dorsal coloration with some specimens paler than others, but otherwise, little variation exists.

##### Etymology.

This species is named *lata*, Latin for broad, for the broad body in this species.

##### Distribution.

This species is known only from the type locality, Brazil, Pará State, Cachimbo (Fig. 78).

##### Type material.

Holotype in USNM, male labeled, “Cachimbo, Para, Brasil, X.1959 M.Alvarenga/ USNMENT01190957 [barcode label]/ HOLOTYPE *Desmopachrialata* Miller, 2021 [red label with black line border].” Paratypes, 21 in USNM and MSBA labeled same as holotype except with different barcode labels (Table 1) and each with “/…PARATYPE *Desmopachrialata* Miller, 2021 [blue label with black line border.”

###### Checklist of *Desmopachriabifurcita* species group

*Desmopachriabifasciata* Zimmermann, 1921 – Brazil (Figs 34, 35)

*Desmopachriabifurcita* sp. nov. – Peru

*Desmopachriabolivari* Miller, 1999 – Bolivia (Figs 36–38)

*Desmopachrialata* sp. nov. – Brazil

*Desmopachriaovalis* Sharp, 1882 – Brazil (Fig. 39)

*Desmopachriavarians* Wehncke, 1877 – Brazil (Figs 40–42) (Braga and Ferreira-Jr. 2018)

### ﻿*Desmopachriaconvexa* species group

**Diagnosis.** This group of *Desmopachria* has an articulable process subapically on the lateral lobe (Fig. 45; Miller 2001; Young 1980, 1981).

**Comments.**Miller (2020) recognized two subgroups based on the size and placement of the articulable process on the lateral lobe and geography. Those with a small­er subapical articulable appendage on the lateral lobe not extending beyond the truncate apex are in the *Desmopachriaconvexa-signata* subgroup, and those with a larger subapical articulable appendage that is leaf-like and extends well beyond the elongate, slender oblique apex of the lateral lobe are in the *Desmopachriaconvexa-convexa* subgroup (Miller 2020). *Desmopachriaconvexa-convexa* species are found in North and Central America and the Caribbean, and *Desmopachriaconvexa-signata* species are found in South America.

#### ﻿ Desmopachria pseudocavia sp. nov.

Taxon classificationAnimaliaColeopteraDytiscidae

D8F195B2-806B-5F8E-BB51-AE4EA4831469

http://zoobank.org/E41416AC-93FD-4951-A2BD-4F063DBCD9F3

Figures 43–45 , 78

##### Type locality.

Venezuela, Bolivar State, Rio Caripito, near Rio Orinoco, 6.58694°N 67.02912°W.

##### Diagnosis.

This species belongs to the *Desmopachriaconvexa-signata* subgroup based on the small articulable, subapical process on the lateral lobe (Fig. 45). Specimens are similar to those of *Desmopachriacavia* Braga & Ferreira Jr. in size, shape, general features, and the presence of a setose depression medially on abdominal sternite VI. However, *Desmopachriapseudocavia* has a distinctly different dorsal color pattern (Fig. 43; Braga and Ferreira-Jr. 2010: fig. 3A) and similar, but differently shaped ***male genitalia.*** In *Desmopachriapseudocavia* the male median lobe terminates in two short rami which are apically rounded and evenly and strongly curved mediad (Fig. 45). In *Desmopachriacavia* these rami are more linear and are apically more distinctly pointed (Braga and Ferreira-Jr. 2010: figs 3D, E). Also, although both species are extremely small diving beetles, *Desmopachriapseudocavia* (TL = 1.1–1.2 mm) are even smaller than *Desmopachriacavia* (TL = 1.3–1.5 mm, Braga and Ferreira-Jr. 2010).

##### Description.

***Measurements.***TL = 1.1–1.2 mm, GW = 0.8 mm, PW = 0.6 mm, HW = 0.4–0.5 mm, EW = 0.2 mm, TL/GW = 1.4–1.5, HW/EW = 2.3–2.4. Body broad, ovoid, laterally broadly curved, lateral margins continuous between pronotum and elytron, body broadest across elytra at midlength of body (Fig. 43).

***Coloration.*** Dorsal surface of head and pronotum yellow. Elytron brown with diffuse, complex maculae, margins of maculae indistinct (Fig. 43). Head appendages, legs, and ventral surfaces yellow.

***Sculpture and structure.*** Head broad, anteriorly rounded, anterior margin of clypeus curved, flattened, margined with conspicuous, continuous flattened narrow bead; surface of head shiny, very finely and sparsely punctate; eyes very large (Fig. 43, HW/EW = 2.3–2.4); antennae short, scape and pedicel relatively large and rounded, flagellomere III long and slender, apically expanded, antennomeres IV-X short and broad, lobed at anterodorsal angle, antennomere XI elongate, apically pointed. Pronotum short, lateral margins short, curved with continuous narrow bead of even width throughout; surface shiny, impunctate to very finely and sparsely punctate. Elytron broad, laterally broadly curved; surface shiny, very finely and sparsely punctate. Prosternum extremely short, longitudinally compressed, medially slightly carinate; prosternal process slender anteriorly, with distinctive, small, sharp medial tubercle, apically short and broad, medially slightly carinate, concave, apically acutely pointed. Metaventrite broad and evenly smoothly convex medially, surface shiny, impunctate; metaventrite wings extremely slender. Metacoxa with medial portion short, < 1/3 length of metaventrite medially, metacoxal lines divergent anteriorly; lateral portion of metacoxa extremely large, anteriorly strongly expanded; surface shiny, impunctate. Metatrochanter large, subequal to length of ventral margin of metafemur; legs otherwise not noticeably modified. Abdomen with surfaces shiny and smooth, very finely and sparsely punctate; abdominal sternite VI with medial longitudinally oval depression with field of setae around margins (Fig. 44).

***Male genitalia.*** Male median lobe in lateral aspect short, slightly curved ventrad; in ventral aspect broad basally, narrowed apically, apex formed as lateral, curved rami with medial rounded emargination (Fig. 45). Lateral lobe in ventral aspect robust, subapically concave on lateral margin, apex truncate, with distinct small subapical articulable process (Fig. 45).

***Sexual dimorphism.*** No obvious sexually dimorphic features were discovered.

***Variation.*** There is substantial variation in the extent and distinctiveness of the dorsal coloration on the elytra (Fig. 43), though the basic pattern seems to be conserved. The humeral and anteromedial maculae and the apical maculae are nearly always present and distinctive. The medial longitudinal macula and lateral maculae are range from nearly absent to vague or indistinct to distinctive.

##### Etymology.

This species is named *pseudocavia*, Latin for resembling *cavia*, for the similarities of this species to *Desmopachriacavia* Braga & Ferreira Jr.

##### Distribution.

This species is known from Venezuela from the states of Amazonas, Anzoategui, Apure, Bolivar, and Monagas (Fig. 78).

##### Type material.

Holotype in MIZA, male labeled, “VENEZUELA: Amazonas State 4°58.838'N, 67°44.341'W; 95m Communidad Caño Gato, on Rio Sipapo; 16.i.2009; leg. Short, Miller, Camacho, Joly, and García VZ09-0116-01X: along stream/ SM0842899 KUNHM-ENT [barcode label]/ HOLOTYPE *Desmopachriapseudocavia* Miller, 2021 [red label with black line border].” Paratypes, 94 specimens, each with paratype label, “…/ PARATYPE *Desmopachriapseudocavia* Miller, 2021 [blue label with black line border]” and different barcode labels (Table 1); 69 in in MIZA, MSBA and SEMC labeled same as holotype; 3 in SEMC labeled, “VENEZUELA: Amazonas State 4°58.845'N, 67°44.345'W; 100m Communidad Caño Gato on Rio Sipapo; sandy stream; 7.i.2006 AS-06-016; leg. A.E.Z. Short/…”; 4 in SEMC labeled, “VENEZUELA: Amazonas State 4°55.849'N, 67°44.645'W, 87m Stream along Río Sipapo 16.i.2009; leg. Short, Garcia, Camacho, Miller, & Joly VZ09-0116-02X: stream habitats/…”; 3 in SEMC labeled, “VENEZUELA: Anzoategui State 9°05.808'N, 64°19.445'W, 236 m River along highway, N. El Tigre 3.ii.2010; leg. A. Short; vegetated backwaters; VZ10-0203-03B/…”; 2 in SEMC labeled, “VENEZUELA: Apure State 7°37.298'N, 69°3.679'W, 83m side road ca. 10 km E. Mantecal leg. Short, Garcia, & Camacho 18.i.2009; marshy area and pool by road; VZ09-0118-02X/…”; 2 in SEMC labeled, “VENEZUELA: Apure State 7°38.660'N, 69°18.004'W, 90m between “La Ye” & Bruzual 19.i.2009; Short, Camacho, & García; VZ09-0118-03X: lagoon/…”; 3 in SEMC labeled, “VENEZUELA: Bolívar State 6.558694°N; 67.02912°W Río Caripito, nr. Río Orinoco, 12.i.2009; leg. Short & Miller VZ09-0112-02A: river margin/…” 7 in SEMC labeled, “VENEZUELA: Monagas State 9°36.591'N, 63°8.295'W, 45 m S. of Maturin; vegetated river/ morichal margin;2.ii.2010; leg. Short & Garcia; VZ10-0202-01B/…”.

### ﻿*Desmopachriaconvexa* species group

#### *Desmopachriaconvexa-convexa* species group

*Desmopachriaaspera* Young, 1981 – Florida, USA

*Desmopachriacenchramis* Young, 1981 – Florida, USA

*Desmopachriachalleti* Miller, 2001 – Colombia

*Desmopachriacircularis* Sharp, 1882 – Guatemala

*Desmopachriaconvexa* – Aubé, 1838 – Eastern USA

*Desmopachriadefloccata* Young, 1981 – Mexico

*Desmopachriaglabella* Young, 1981 – Cuba

*Desmopachriagrana* – LeConte, 1855 – Eastern USA

*Desmopachriaisthmia* Young, 1981 – Panama

*Desmopachrialaesslei* Young, 1981 – Jamaica

*Desmopachrialewisi* Young, 1981 – Jamaica

*Desmopachriamajuscula* Young, 1990 – Guatemala

*Desmopachriamortimer* Miller, 2021 – Costa Rica

*Desmopachriatarda* Spangler, 1973 – Cuba

#### Desmopachriaconvexa-signata species group

*Desmopachriacavia* Braga & Ferreira Jr., 2010 – Brazil

*Desmopachriamanco* Miller, 2021 – Guyana

*Desmopachriamanus* Braga & Ferreira Jr., 2010 – Brazil

*Desmopachriapilosa* Miller, 2005 – Peru

*Desmopachriapseudocavia* sp. nov. – Venezuela

*Desmopachriasignata* Zimmermann, 1921 – Brazil

*Desmopachriasignatoides* Miller, 2001 – Bolivia

*Desmopachriavarzeana* Braga & Ferreira Jr., 2010 – Brazil

### ﻿*Desmopachrianitida* species group

**Diagnosis.** This species group is characterized by bifid lateral lobes (e.g., Fig. 50). In some species, the median lobe is relatively simply, apically narrowly bifid with the apices sharply pointed. In others, it is extremely complex, apically modified, with various branches and structures. This group includes some of the most complex, convoluted male genitalia in Dytiscidae (Miller and Wolfe 2018).

**Comments.** This species-group was originally in *Desmopachria* sensu stricto (Young 1980), with only one species. Additional species were added by Young (1986; 1989; 1990a), Miller (1999; 2005), and Braga and Ferreira-Jr. (2014). Other previously described species were moved into the group by Miller (2001). Miller and Wolfe (2018) described the species including new ones and reviewed the taxonomy of the group, including concerns about the species-group placement of newly described species by Makhan (2012, 2015).

#### ﻿ Desmopachria wolfei sp. nov.

Taxon classificationAnimaliaColeopteraDytiscidae

7072770B-CC3C-570C-8A2A-95B8C6E4EDFC

http://zoobank.org/854B6B76-1805-4601-8192-8772F6371F9E

Figures 46–50 , 79

##### Type locality.

Venezuela, Bolivar State, Rio Aponwao at Highway 10, 5°50'49.2"N, 62°28'2.4"W.

##### Diagnosis.

This species belongs to the *Desmopachrianitida* group sensu Miller (2001) because of presence of deeply bifid male lateral lobes (Fig. 50). Within the group, the species has a bilaterally symmetrical, but extremely complicated male median lobe that is very unique in shape, medially distinctly constricted, apically broadly truncate, and with an apicomedial emargination, among other shape characteristics (Figs 47–50). Dorsal coloration is simple with the head and pronotum orange and elytron brown.

##### Description.

**Figures 46–50.**
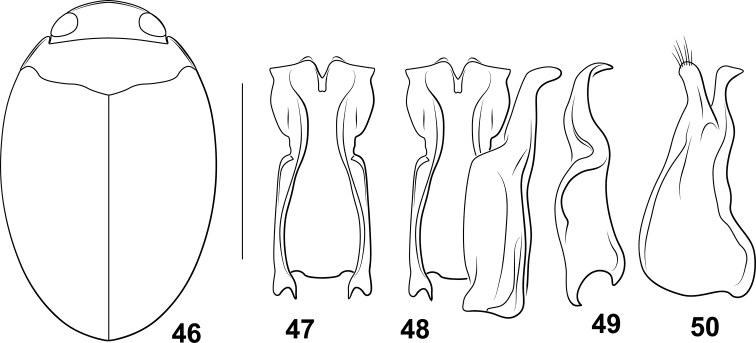
*Desmopachriawolfei.***46** habitus **47** male median lobe, right lateral aspect **48** male median lobe, ventral aspect **49** male median lobe and right lateral lobe, ventral aspect **50** right lateral lobe, right lateral aspect. Scale bar: 1.0 mm.

***Measurements.***TL = 1.8–1.9 mm, GW = 1.2–1.3 mm, PW = 0.9–1.0 mm, HW = 0.6–0.7 mm, EW = 0.3 mm, TL/GW = 1.4–1.5, HW/EW = 1.9–2.0. Body broad, elongate oval, laterally broadly curved, lateral margins continuous between pronotum and elytron, body broadest across elytra at midlength of body (Fig. 46).

***Coloration*** (Fig. 46). Dorsal surface of head and pronotum evenly pale orange. Elytron dark orange, darker than surface of pronotum. Head appendages, legs and ventral surfaces orange to dark orange or orange-red.

***Sculpture and structure.*** Head broad; anterior margin of clypeus evenly curved, flattened, margined with conspicuous, continuous narrow bead; surface of head shiny, finely and sparsely punctate; eyes large (HW/EW = 1.9–2.0); antennae short, scape and pedicel relatively large and rounded, flagellomere III long and slender, apically expanded, antennomeres IV-X short and broad, lobed at anterodorsal angle, antennomere XI elongate, apically pointed. Pronotum short, lateral margins short, sublinear with continuous narrow bead, slightly wider medially; surface shiny, impunctate medially, punctation denser along anterior and posterior margins, punctation fine. Elytron moderately broad, laterally broadly curved; surface shiny, more coarsely and evenly punctate than pronotum, punctation distinctive and prominent. Prosternum extremely short, longitudinally compressed, medially slightly carinate; prosternal process slender anteriorly, with distinctive, small medial tubercle, apical blade short and broad, basally transversely carinate, medially concave, apically broadly pointed. Metaventrite broad and evenly smoothly convex medially, surface shiny, very finely and sparsely; metaventrite wings extremely slender. Metacoxa with medial portion short, < 1/3 length of metaventrite medially, metacoxal lines divergent anteriorly; lateral portion of metacoxa extremely large, anteriorly strongly expanded; surface shiny, very finely and sparsely punctate. Metatrochanter large, subequal to length of ventral margin of metafemur; legs otherwise not noticeably modified. Abdomen with surfaces shiny and smooth, very finely and sparsely punctate.

***Male genitalia.*** Male genitalia complex; median lobe elongate in lateral aspect, broad basally, medially constricted, broadly sinuate, apex strongly curved dorsal, apically pointed (Fig. 47); median lobe in ventral aspect very broad, lateral margins subparallel in basal half, with distinct constriction medially due to distinct lateral emarginations, apical half with lateral margins curved to apicolateral angle, apex very broadly truncate with distinct medial emargination (Figs 48, 49). Lateral lobe in broad basally, apically conspicuously bifurcate, dorsal branch apically broadly rounded, ventral branch apically truncate with dorsal corner acutely pointed dorsad (Fig. 50).

***Sexual dimorphism.*** No obvious sexual dimorphic features were discovered.

***Variation.*** Some specimens are variously paler or darker in coloration than others.

##### Etymology.

This distinctive species is named *wolfei* for G.W. Wolfe, gifted coleopterist, exemplary water beetle biologist, exceptionally fine husband and father, and the author’s dear friend for many years.

##### Distribution.

This species is known only from Bolivar State, Venezuela (Fig. 79).

##### Type material.

Holotype in MIZA, male labeled, “VENEZUELA: Bolivar State 5°50'49.2"N, 62°28'2.4"W, 1340 m Rio Aponwao @ Hwy 10 31.vii.2008; leg. A.Short, M. García AS-08-060a; small vegetated pool/ SM0827530 KUNHM-ENT [barcode label] / HOLOTYPE *Desmopachriawolfei* Miller, 2021 [red label with black line border].” Paratypes, 23; 9 in MIZA and SEMC labeled same as holotype except with “… PARATYPE *Desmopachriawolfei* Miller, 2021 [blue label with black line border]” and different barcode labels (see Table 1); 14 in SEMC labeled, “VENEZUELA: Bolivar State 4°49.944'N, 61°3.813'W, 890m ca. 25 km S. San Francisco 3.viii.2008; A.Short & M. García AS-08-069; large marsh/...”

###### Checklist of species in the *Desmopachrianitida* species group

*Desmopachriaannae* Miller, 2005 – Bolivia

*Desmopachriaanauine* Braga & Ferreira-Jr., 2018 – Brazil

*Desmopachriaaschnae* Makhan, 2012 – Suriname. This species is hereby placed into the *Desmopachrianitida* species group based on published figures of bifid lateral lobes (Makhan 2012: fig. 4).

*Desmopachriabalionota* Miller, 2005 – Peru, Brazil (Braga and Ferreira-Jr. 2010, 2014).

*Desmopachriacurseenae* Miller & Wolfe, 2018 – Suriname

*Desmopachriadarlingtoni* Young, 1989 – Jamaica, Cuba, Haiti, Colombia

*Desmopachriadelongi* Miller & Wolfe, 2018 – Suriname

*Desmopachriadraco* Miller, 1999 – Bolivia, Brazil (Braga and Ferreira-Jr. 2010)

*Desmopachriagingerae* Miller & Wolfe, 2018 – Venezuela

*Desmopachriagranoides* Young, 1986 – Brazil (Braga and Ferreira-Jr. 2014), Bolivia, Suriname, Venezuela, Trinidad

*Desmopachriagyrationi* Miller & Wolfe, 2018 – Guyana

*Desmopachriahardyae* Miller & Wolfe, 2018 – Guyana

*Desmopachriakemptonae* Miller & Wolfe, 2018 – Venezuela

*Desmopachrialeptophallica* Braga & Ferreira-Jr., 2014 – Brazil

*Desmopachrialiosomata* Young, 1986 – Brazil

*Desmopachrialloydi* Miller & Wolfe, 2018 – Bolivia

*Desmopachriamargarita* Young, 1990 – Panama, Brazil? (Braga and Ferreira-Jr. 2014).

*Desmopachrianitida* Babington, 1841 – Brazil

*Desmopachrianitidoides* Young, 1990 – Paraguay

*Desmopachriaphacoides* Guignot, 1950 – Paraguay, Bolivia

*Desmopachriapsarammo* Miller, 1999 – Bolivia

*Desmopachriarhea* Miller, 1999 – Bolivia

*Desmopachriasinghae* Miller & Wolfe, 2018 – Venezuela

*Desmopachriasubnotata* Zimmermann, 1921 – Brazil (Braga and Ferreira-Jr. 2010).

*Desmopachriasubtilis* Sharp, 1882 – Brazil

*Desmopachriavohrae* Miller & Wolfe, 2018 – Venezuela

*Desmopachriawolfei* sp. nov. – Venezuela

*Desmopachriazelota* Young, 1990 – Brazil

### ﻿*Desmopachriaportmanni* species group

**Diagnosis.** The *Desmopachriaportmanni* group is well characterized morphologically by males with a bifid prosternal process with a deep medial pit (Miller 2001; Young 1980; 1995). Some are dorsally distinctly iridescent (the *Desmopachriaportmanni-aldessa* subgroup), but others are not (the *Desmopachriaportmanni-portmanni* subgroup). Other species in *Desmopachria* are iridescent but lack the forked, pitted process and are not included in the *Desmopachriaportmanni* group (see Miller 2001) including *Desmopachriadivergens* sp. nov. (described above). There are also additional species that have similar genitalia (simple, elongate median lobe, elongate lateral lobes with dense medial series of setae (such as *Desmopachrialineata* sp. nov., described above, Fig. 4) but do not have a bifid prosternal process. Careful phylogenetic work needs to be conducted to determine the relationships among these various taxa.

**Comments.**Young (1980) placed those taxa with a forked prosternal process and a deep medial pit into two groups, those with distinct dorsal iridescence (the subgenus Desmopachria (Pachiridis) Young) and those without dorsal iridescence (the subgenus Desmopachria (Portmannia) Young). Given the exceptional uniqueness of the forked prosternal feature, Miller (2001) found it a compelling synapomorphy for these species and lumped them together into one group, the *Desmopachriaportmanni* species group. Another group, the *Desmopachriaubangoides* species group sensu Miller (2001) (= Desmopachria (Hintonia) Young) exhibits dorsal iridescence, like some species in the *Desmopachriaportmanni* group. These species seem rather different in other ways since they lack the forked prosternal process and have male genitalia that are not consistent with the relatively simple structures present in the *Desmopachriaportmanni* group. Their genitalia are relatively more complex and differently shaped. Also, the anterior clypeal margin is sexually dimorphic. In males it is strongly modified, flattened, and upturned, whereas in females it is beaded, but not so conspicuously modified. For this reason, they are still regarded here as a separate species group (the *Desmopachriaubangoides* species group). Prior to this paper 23 species were assigned to the *Desmopachriaportmanni* group (Nilsson 2016).

Within the *Desmopachriaportmanni* group (Desmopachria (Portmannia) Young), Young (1980) recognized two subgroups based on coloration and punctation, but when later revising the subgenus (Young 1995) he mentioned the two groups but did not assign specimens to them and seemed to have abandoned a more formal recognition of them. However, there seems to be some utility in recognizing two groups, those that are iridescent versus those that are not, but which are often dorsally maculate. The two subgroups are here recognized as the *Desmopachriaportmanni-aldessa* subgroup and the *Desmopachriaportmanni-portmanni* subgroup (see below).

Young (1995) found members of the *Desmopachriaportmanni* group (as Desmopachria (Portmannia) Young to be found mainly in higher elevations rather than lowland tropics. This may be true in North and Central American species but does not appear to be entirely consistent with northern South American species.

Although somewhat larger than many other *Desmopachria*, these are still tiny diving beetles occurring in a variety of habitats, but especially in tropical forest pools and streams. It should be noted that no specific adaptive significance is known for either the uniquely forked and deeply pitted male prosternal process nor the dorsal iridescence of specimens of both sexes of many species. Young (1995) thought the forked prosternal process might be a “…small suction organ during copulation, or is a device for pheromone retention,” but these hypotheses have not been critically examined, and each seem dubious.

As with *Desmopachria* in general (Braga and Ferreira-Jr. 2010, 2011, 2014; Gustafson and Miller 2012; Miller 1999, 2001, 2005), writing a key to species in this group is extremely difficult. It is much easier to simply compare diagnostic features of the male genitalia with illustrations to make species determinations. Other characters, such as degree and type of punctation amount of iridescence, and size are somewhat variable between species are useful as diagnostic features. For this reason, illustrations are emphasized here rather than a dichotomous key. Diagnostic descriptions of punctation should also be examined for identifications.

### ﻿*Desmopachriaportmanni-aldessa* subgroup

**Diagnosis.** Within the *Desmopachriaportmanni* species-group, these species have the dorsal surface iridescent. In some species the amount of iridescence is more limited but is distinct at least medially on the elytron using standard microscopy lighting.

**Comments.** These are species of northern South America. They are difficult to identify and need additional work to clarify species limits. It seems likely that there are numerous additional species given the narrow geographic ranges of the known species and the ambiguity of some species limits. Specifically species including and near *Desmopachriaaurea* Young need some examination. These are species with male median lobes that are short with varying degrees of lateral margin curvature and apical truncation.

#### ﻿ Desmopachria angulata sp. nov.

Taxon classificationAnimaliaColeopteraDytiscidae

A989B6AB-0A32-5F29-9CBA-0E06B71E3692

http://zoobank.org/29C963D1-E292-41B1-91E1-B0076283904

Figures 51–53 , 79

##### Type locality.

Suriname, Sipaliwini District, Camp 2 on Sipaliwini River, large forest stream, 2°10.937'N 56°47.235°W.

##### Diagnosis.

**Figures 51–71.**
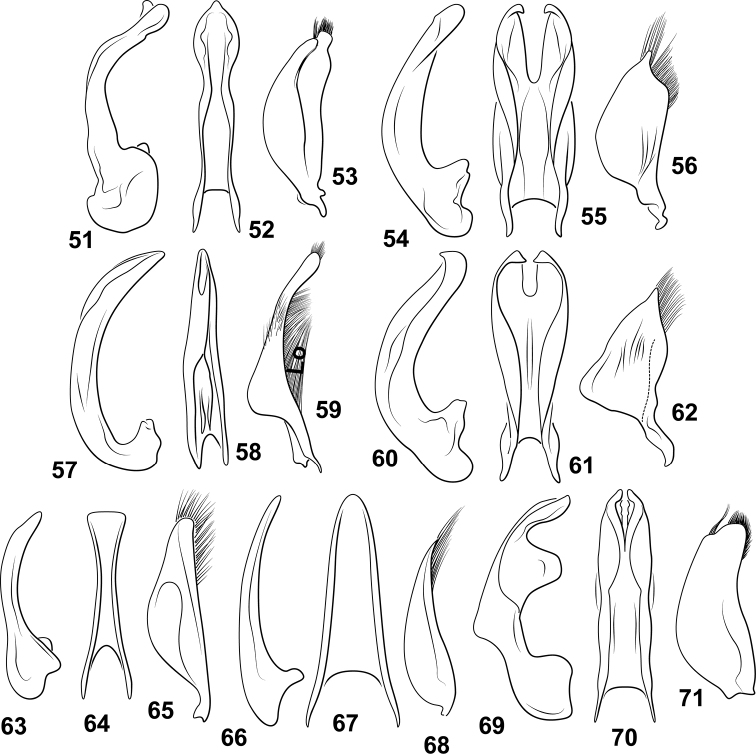
*Desmopachria* species. (**51–53**) *Desmopachriaangulata***51** median lobe, right lateral aspect **52** median lobe, ventral aspect **53** right lateral lobe, right lateral aspect (**54–56**) *Desmopachriaemarginata***54** median lobe, right lateral aspect **55** median lobe, ventral aspect **56** right lateral lobe, right lateral aspect (**57–59**) *Desmopachriaimparis***57** median lobe, right lateral aspect **58** median lobe, ventral aspect **59** right lateral lobe, right lateral aspect (**60–62**) *Desmopachriaimpunctata***60** median lobe, right lateral aspect **61** median lobe, ventral aspect **62** right lateral lobe, right lateral aspect (**63–65**) *Desmopachriatruncata***63** median lobe, right lateral aspect **64** median lobe, ventral aspect **65** right lateral lobe, right lateral aspect (**66–68**) *Desmopachriabisulcata***66** median lobe, right lateral aspect **67** median lobe, ventral aspect **68** right lateral lobe, right lateral aspect (**69–71**) *Desmopachriairregulara***69** median lobe, right lateral aspect **70** median lobe, ventral aspect **71** right lateral lobe, right lateral aspect.

This species is dorsally iridescent. The punctation on the pronotum is very fine and sparse. The punctation on the elytron is dual, with some large and some small and interspersed among the larger ones. The male genitalia are distinctive with the median lobe elongate, slender and distinctly angulate medially with the apex rounded and with a distinct subapical lobe on the ventral margin (Fig. 51). The lateral lobe is broad medially with the apex lobate and narrowly rounded with a small, dense series of setae (Fig. 53).

##### Description.

***Measurements.***TL = 2.0–2.2 mm, GW = 1.5–1.6 mm, PW = 1.1–1.2 mm, HW = 0.4–0.5 mm, EW = 0.4 mm, TL/GW = 1.4–1.5, HW/EW = 1.8–2.0. Body very broad, laterally rounded, lateral margins slightly discontinuous between pronotum and elytron; dorsoventrally compressed.

***Coloration.*** Head and pronotum evenly orange, slightly iridescent. Elytron evenly brownish orange, iridescent. Ventral surface of head, prosternum, head appendages, and pro- and mesolegs yellow, other ventral surfaces and metalegs darker orange, lateral portion of metacoxa iridescent.

***Sculpture and structure.*** Head broad, short; anterior margin of clypeus finely margined with continuous flattened narrow bead; surface of head shiny, punctation extremely fine, evenly distributed; eyes large (HW/EW = 1.8–2.0); antennae short, scape and pedicel relatively large and rounded, flagellomere III long and slender, apically expanded, antennomeres IV-X short and broad, antennomere XI elongate, apically pointed. Pronotum short, lateral margins short, broadly curved with continuous narrow bead; surface shiny, punctation fine, slightly irregular in size, few larger punctures; posterior margin sinuate. Elytron broad, laterally broadly curved; surface shiny, conspicuously punctate, punctures dual with fine and large interspersed. Prosternum extremely short, longitudinally compressed, medially slightly carinate; prosternal process in male very slender anteriorly, with low, indistinct medial tubercle, bifid apically with deep medial pit, in female slender anteriorly, with distinctive, small medial tubercle, apically short and broad, medially slightly carinate, apically acutely pointed. Metaventrite broad and evenly smoothly convex medially, surface shiny, impunctate; metaventrite wings extremely slender. Metacoxa with medial portion short, < 1/3 length of metaventrite medially, metacoxal lines sinuate, strongly divergent anteriorly; lateral portion of metacoxa extremely large, anteriorly strongly expanded; surface shiny, impunctate. Metatrochanter very large, subequal to length of ventral margin of metafemur; legs otherwise not noticeably modified. Abdomen with surfaces shiny and smooth, very finely and sparsely punctate.

***Male genitalia.*** Male median lobe in lateral aspect with basal portion broad and rounded, apically long and slender, medially conspicuously bent, with distinctive subapical lobe on ventral surface (Fig. 51); in ventral aspect elongate, slender, lateral margins sinuate, subapically broadly and roundly expanded, apex broadly pointed (Fig. 52). Lateral lobe broad, ventrally broadly rounded, apically lobate with subapical lobe on lateral surface, apices of lobes with dense fringe of setae (Fig. 53).

***Sexual dimorphism.*** Male pro- and mesotarsomeres I-III slightly more broadly expanded and with ventral adhesive setae. Male and female prosternal processes different as in all *Desmopachriaportmanni* group species.

***Variation.*** Some specimens have the dorsal punctation more or less dense than described above. Also, coloration varies in intensity, though the head and pronotum are always paler than the elytra.

##### Etymology.

This species is named *angulata*, Latin for angled, for the strongly angulate male median lobe in lateral aspect (Fig. 51).

##### Distribution.

This species is known from Region 6, Guyana and Sipaliwini District, Suriname (Fig. 79).

##### Type material.

Holotype in NZCS, male labeled, “SURINAME: Sipaliwini District 2°10.973'N, 56°47.235'W, 210m Camp 2, on Sipaliwini River; Short & Kadosoe; large forest stream 28–29.viii.2010; SR10-0828-02A 2010 CI-RAP Survey/ SEMC0913662 KUNHM-ENT [barcode label]/ HOLOTYPE *Desmopachriaangulata* Miller, 2021 [red label with black line border].” Paratypes, 144 total in NZCS and SEMC (from Suriname), CSBD and SEMC (from Guyana), and MSBA (various), each with different barcode labels (Table 1) and “…PARATYPE *Desmopachriaangulata* Miller, 2021 [blue label with black line border]; 17 labeled same as holotype; 7 labeled, “GUYANA: Region 6 4°09.241'N,58°10.627'W, 109 m Upper Berbice, Basecamp 1 puddles along road leg. Short, Salisbury, La Cruz 24.ix.2014; GY14-0924-02A/…”; 1 labeled same, except, “…4°09.143'N, 58°11.207'W, 105 m…margins of creek…22.iv.2014; GY1-0921-03H/…”; 1 labeled same, except, “…4°09.289'N, 58°11.717'W, 96 m…side pools of creek…21.iv.2014; GY1-0921-01B/…”; 1 labeled same, except, “…4°09.126'N, 58°12.274'W, 73 m…3km W. of Basecamp 1; pooled up creek……[no date]; GY1-0923-01A/…”; 2 labeled, “GUYANA: Region 6 4°09.136'N, 58°11.365'W, 106 m Upper Berbice, ca 1.1km W of Basecamp 1; stream detrital pools leg. Short, Salisbury, La Cruz 23.ix.2014; GY14-0923-02A/…”; 4 labeled, “GUYANA: Region 6 4°09.143'N, 58°11.207'W, 105 m Upper Berbice, c. 1km W of Basecamp 1; small sandy stream leg. Short, Salisbury, La Cruz 21.ix.2014; GY14-0921-03A/…”; 2 labeled same, except, “…side pools of creek…22.ix.2014; GY14-0921-03G/…”; 11 labeled, “SURINAME: Sipaliwini District 2°21.776'N, 56°41.861'W, 237 m Camp 3, Wehepai leg. Short & Kadosoe; pooled up detrital creek 3.ix.2010; SR10-0903-01A 2010 CI-RAP Survey/…”; 1 labeled same, except, “…sandy forest creek 4-6.ix.2010; SR10-0904-01A/…”; 4 labeled, “SURINAME: Sipaliwini District 2°10.973'N, 56°47.235'W, 210 m Camp 2, on Silpaliwini [sic] River; Short & Kadosoe; small detrital stream 28.viii.2010; SR10-0828-03A 2010 CI-RAP Survey/…”; 6 labeled same, except, “…Sipaliwini…inselberg…29-30.viii.2010; SR10-0829-01A…”; 7 labeled same, except, “…sandy forest creek w/detritus; SR10-0831-01B 31.viii.2010…”; 5 labeled same, except, “…forest creek 31.viii.2010; SR10-0831-01A…”; 9 labeled, “SURINAME: Sipaliwini District N 2.47700°N, 55.62941°W, 275 m Camp 1, Upper Palumeu leg. A Short; Flight Intercept Trap 10-16.iii.2012; SR12-0310-TN1 2012 CI-RAP Survey/…”; 1 labeled same, except, “… Upper Palumeu River…large detrital pools 10-12.iii.2012; SR12-0310-01A…”; 58 labeled, “SURINAME: Sipaliwini District 2°10.521'N, 56°7.244'W, 228 m on Kutari River; leg. Short & Kadosoe; forested swamp 19.viii.2010; SR10-0819-01A Camp 1; 2010 CI-RAP Survey/…”; 1 labeled same, except, “…forest stream…SR10-0819-02A…”; 5 labeled, “SURINAME S2005-13 03May2005. Palumeu off Tapanahoni R. just upstream fr S2005-10 N03.36951 W055.43654 Wolfe-Hiwat-class.”

#### ﻿ Desmopachria emarginata sp. nov.

Taxon classificationAnimaliaColeopteraDytiscidae

434B8CDF-CF41-569B-986E-A6BFED2BDA3C

http://zoobank.org/C3F3E5FA-79B6-4F74-9A03-0C32E955606B

Figures 54–56 , 80

##### Type locality.

Suriname, Sipaliwini District, Tafelberg Summit, near Augustus Creek Camp, pond on trail into Arrowhead Basin, 3°55.600'N, 56°11.300'W.

##### Diagnosis.

This species includes some of the largest specimens in the group (TL = 2.0–2.2 mm). The elytra are more distinctly punctate than most species with distinctive dual punctation. Also, the elytra are very noticeably iridescent in most specimens, but a series from Raleighfallen Nature Preserve, Sipaliwini District, Suriname (NZCS) are less conspicuously iridescent. These specimens are similar in size, punctation and male genitalia, however. The male genitalia are most similar to *Desmopachriaimpunctata*, but the apical emargination in the median lobe is relatively deeper and in lateral aspect the apex is not distinctly hooked ventrad (Figs 54, 55 vs. Figs 60, 61). Also, *Desmopachriaemarginata* are larger (TL = 2.0–2.2 mm) than *Desmopachriaimpunctata* (TL = 1.8–1.9 mm).

##### Description.

***Measurements.***TL = 2.0–2.2 mm, GW = 1.4–1.5 mm, PW = 1.1–1.2 mm, HW = 0.7–0.8 mm, EW = 0.4–0.5 mm, TL/GW = 1.4–1.5, HW/EW = 1.8–1.9. Body very broadly oval, laterally broadly curved, lateral margins nearly continuous between pronotum and elytron.

***Coloration.*** Head orange. Pronotum pale orange, in a majority of specimens paler in color than head or elytron. Elytron evenly brownish orange, strongly iridescent. Ventral surface of head, prosternum, head appendages, and pro- and mesolegs pale orange, other ventral surfaces and metalegs orange.

***Sculpture and structure.*** Head broad, short; anterior margin of clypeus broadly curved, flattened, finely margined with conspicuous, continuous narrow bead, particularly evident medially; surface of head shiny, extremely finely and sparsely punctate; eyes large (HW/EW = 1.8–1.9); antennae short, scape and pedicel relatively large and rounded, flagellomere III long and slender, apically expanded, antennomeres IV-X short and broad, antennomere XI elongate, apically pointed. Pronotum short, lateral margins short, slightly curved with continuous marginal bead; surface shiny, impunctate medially, but laterally and posteriorly with fine punctation. Elytron broad, laterally broadly curved and rounded; surface shiny, prominently punctate, punctures dual, some larger, some smaller, interspersed. Prosternum extremely short, longitudinally compressed, medially carinate; prosternal process in male very slender anteriorly, with low, indistinct medial tubercle, bifid apically with deep medial pit, in female slender anteriorly, with distinctive, small medial tubercle, apically short and broad, medially slightly carinate, apically acutely pointed. Metaventrite broad and evenly smoothly convex medially, surface shiny, impunctate or with few extremely minute punctures laterally; metaventrite wings extremely slender. Metacoxa with medial portion short, ~ 1/3 length of metaventrite medially, metacoxal lines slightly sinuate, divergent anteriorly; lateral portion of metacoxa extremely large, anteriorly strongly expanded; surface shiny, impunctate or with few extremely fine punctures. Metatrochanter very large, subequal to length of ventral margin of metafemur; legs otherwise not noticeably modified. Abdomen with surfaces shiny and smooth, impunctate, some specimens with few very fine punctures.

***Male genitalia.*** Male median bilaterally symmetrical, in lateral aspect elongate, evenly broad, evenly and broadly curved on both dorsal and ventral margins, apex linear and apically broadly rounded (Fig. 54); in ventral aspect broad basally, apically broad, lateral margins irregularly sublinear, apex broad, medially deeply emarginate, emargination ~ 2/5 length, apical branches each apex with small process extending medially (Fig. 55). Lateral lobe very broad medially, ventral margin broadly rounded, apex narrowed to small lobe, with series of long setae apically and along apicodorsal margins (Fig. 56).

***Sexual dimorphism.*** Male pro- and mesotarsomeres I-III slightly more broadly expanded and with ventral adhesive setae. Male and female prosternal processes different as in all *Desmopachriaportmanni* group species.

***Variation.*** Specimens vary in intensity of coloration and degree of difference between head, pronotum and elytron, but not strongly so.

##### Etymology.

This species is named *emarginata*, Latin for the apical emargination in the male median lobe.

##### Distribution.

This species is known from localities in Guyana, Suriname, and Venezuela (Fig. 80).

##### Type material.

Holotype in NZCS, male labeled, “SURINAME: Sipaliwini District 3°55.600'N, 56°11.300'W, 600m CSNR: Tafelberg Summit, nr Augustus Creek Camp, pond on trail into Arrowhead basin leg. Short & Bloom; 16.viii.2013 SR13-0816-02A/ SEMC0930719 KUNHM-ENT [barcode label]/ HOLOTYPE *Desmopachriaemarginata* Miller, 2021 [red label with black line border].” Paratypes, 153 specimens in CSBD, MIZA, MSBA NCZS, and SEMC each with paratype label, “…/ PARATYPE *Desmopachriaemarginata* Miller, 2021 [blue label with black line border]” and different barcode labels (Table 1); 43 labeled same as holotype; 2 in SEMC labeled, “GUYANA: Region IX 2°09.557'N, 59°17.569'W, 268m along road to Parabara forest pools near Mushai Wao leg. Short, Isaacs, Salisbury 1.xi.2013; GY13-1101-02A/…”; 3 in SEMC labeled, “GUYANA: Region IX 2°47.417'N, 59°53.986'W, 113m Kusad Mts., Taraara Wao creek margin & isolated side pools leg. Short, Isaacs, Salisbury 28.x.2013; GY13-1028-01A/…”; 2 in SEMC labeled, “GUYANA: Region IX 2°06.311'N, 59°14.072'W, 267m Parabara, N. side of river small detrital pool in forest leg. A.E.Z. Short; 2.xi.2013 GY13-1103-01A/…”; 3 in SEMC labeled, “GUYANA: Region 6 4°45.301'N, 58°00.404'W, 49 m Upper Berbice Basecamp 2 shallow detrital pool in forest draining into creek; leg. Short, Salisbury, La Cruz; 26.ix.2014 GY14-0926-01A/…”; 9 in SEMC labeled, “GUYANA: Region XIII [sic] 4.988650°N, 59.57890°W, 427 m Chenapau Village, trail between airstrip & town; large pool with lots of detritus; leg. A. Short 14.iii.2014; GY14-0314-03A/…”; 33 in CSBD and SEMC labeled, “GUYANA: Region XIII [sic] 5°10.514'N, 59°28.970'W, 440 m Kaieteur Nat’l Park, trail by guest house; small forest stream leg. A. Short; 15.iii.2014 GY14-0315-03A/…”; 7 in SEMC labeled, “GUYANA: Region XIII [sic]4.98650°N, 59.57890°W, 427 m Chenapau village, between airstrip & town, small pools along trail leg. Short & Baca; 10.iii.2014 GY14-0310-01A/…”; 4 in SEMC labeled, “SURINAME: Sipaliwini District 3°47.479'N, 56°08.968'W, 320m CSNR: near Kappel airstrip forested stream & stream pools leg. Short & Bloom; 24.viii.2013 SR13-0824-03A/…”; 15 in NZCS and SEMC labeled, “SURINAME: Sipaliwini District 3°53.942'N, 56°10.849'W, 733m CSNR: Tafelberg Summit, nr. Caiman Crk Camp; dipnetting by stream margins: leg. Bloom 20.viii.2013, SR13-0820-02A/…”; 1 in SEMC labeled, “SURINAME: Sipaliwini District 2°10.973'N, 56°47.235'W, 210 m Camp 2, on Silpaliwini [sic] River; Short & Kadosoe; small detrital stream 28.viii.2010; SR10-0828-03A 2010 CI-RAP Survey/…”; 20 in NZCS labeled, “SURINAME: Sipaliwini District 3°55.600'N, 56°11.300'W, 600m CSNR: Tafelberg Summit, nr Augustus Creek Camp, pond on trail into Arrowhead basin leg. Short & Bloom; 16.viii.2013 SR13-0816-02A/…”; 7 in SEMC labeled, “SURINAME: Sipaliwini District 04°40.910'N, 56°11.138'W, 78 m Raleighfallen Nature Reserve Voltzberg trail; Margin of stream leg. C. Maier, V. Kadosoe 30.vii.2012; SR12-0730-01A/…”; 3 in MIZA and SEMC labeled, “VENEZUELA: Bolívar State 4°28.233'N, 61°35.559'W, 867 m Gran Sabna, Pauji: Esmeraldes 16.vii.2010;leg. Short, Tellez, Arias detrital pools by forested stream VZ10-0716-02A/…”; 1 in SEMC labeled, “VENEZUELA: Bolívar State 5°50'49.2"N, 61°28'2.4"W, 1340 m Rio Aponwao @ Hwy 10 31.vii.2008; leg. A.Short, M. García AS-08-060a; small vegetated pool/…”.

#### ﻿ Desmopachria imparis sp. nov.

Taxon classificationAnimaliaColeopteraDytiscidae

4DF75A79-6CFB-5AD1-B9D5-4814C8FE019F

http://zoobank.org/27F2D74C-8D9B-4ACF-98C2-5C13309BE0F5

Figures 57–59 , 79

##### Type locality.

Guyana, Region IX, Parabara, trail to mines, 2°05.095'N, 59°14.174'W.

##### Diagnosis.

This species is unique because of the asymmetrical shape of the male median lobe. In ventral aspect the median lobe is distinctly asymmetrical, though not strongly so (Fig. 58). However, this is the only known species of the *Desmopachriaportmanni* group with an asymmetrical male median lobe. Punctation on the elytron is dual with some small punctures interspersed among numerous larger punctures. The elytral surface is otherwise shiny with some opalescent iridescence evident. Externally, among northern South American species this one is similar to *Desmopachrialineata* sp. nov., but that species has distinctive linear series of punctures on the elytra and a differently shaped male median lobe (Figs 3, 4).

##### Description.

***Measurements.***TL = 2.1–2.2 mm, GW = 1.5–1.6 mm, PW = 1.1–1.2 mm, HW = 0.7–0.8 mm, EW = 0.4 mm, TL/GW = 1.4, HW/EW = 1.8–1.9. Body broadly oval, laterally broadly curved, lateral margins approximately continuous between pronotum and elytron.

***Coloration.*** Head and pronotum evenly yellow. Elytron evenly brownish orange, iridescent, especially apically. Ventral surface of head, prosternum, head appendages, and pro- and mesolegs yellow, other ventral surfaces and metalegs darker orange.

***Sculpture and structure.*** Head broad, short; anterior margin of clypeus broadly curved, flattened, margined with conspicuous, continuous flattened narrow bead; surface of head shiny, extremely finely and sparsely punctate; eyes large (HW/EW = 1.8–1.9); antennae short, scape and pedicel relatively large and rounded, flagellomere III long and slender, apically expanded, antennomeres IV-X short and broad, antennomere XI elongate, apically pointed. Pronotum short, lateral margins short, slightly curved with continuous narrow bead; surface shiny, extremely finely and sparsely punctate, posterior margin sinuate. Elytron broad, laterally broadly curved and rounded; surface shiny, punctate, punctures dual, mostly larger, interspersed with smaller ones. Prosternum extremely short, longitudinally compressed, medially carinate; prosternal process in male very slender anteriorly, with low, indistinct medial tubercle, bifid apically with deep medial pit, in female slender anteriorly, with distinctive, small medial tubercle, apically short and broad, medially slightly carinate, apically acutely pointed. Metaventrite broad and evenly smoothly convex medially, surface shiny, impunctate medially, shallowly and minutely punctate laterally and posteromedially; metaventrite wings extremely slender. Metacoxa with medial portion short, < 1/3 length of metaventrite medially, metacoxal lines slightly sinuate, divergent anteriorly; lateral portion of metacoxa extremely large, anteriorly strongly expanded; surface irrorate, somewhat opalescent. Metatrochanter very large, subequal to length of ventral margin of metafemur; legs otherwise not noticeably modified. Abdomen with surfaces shiny and smooth, somewhat opalescent, very finely and sparsely punctate.

***Male genitalia.*** Male median lobe slightly but distinctly asymmetrical; in lateral aspect long, evenly broad, evenly and broadly curved on both dorsal and ventral margins, apex narrowly rounded (Fig. 57); in ventral aspect asymmetry evident, moderately broad basally, apically narrowed to narrowly rounded apex, lateral margins irregularly sublinear on each side, but different in shape on each side, medial groove directed primarily to right of central line (Fig. 58). Lateral lobe broad basally, abruptly narrowed and slender for most of apical length, apex slightly broadened and broadly rounded, with dense long series of setae along dorsal margin medially and with field of short setae mediolaterally, apex with series of short setae (Fig. 59).

***Sexual dimorphism.*** Male pro- and mesotarsomeres I-III slightly more broadly expanded and with ventral adhesive setae. Male and female prosternal processes different as in all *Desmopachriaportmanni* group species.

***Variation.*** No significant variation was discovered among the few specimens examined.

##### Etymology.

This species is named *imparis*, Latin for uneven, referring to the asymmetrical male median lobe.

##### Distribution.

This species is known from one locality in Region IX, Guyana (Fig. 79).

##### Type material.

Holotype in CSBD, male labeled, “GUYANA: Region IX 2°05.095'N, 59°14.174'W, 250m Parabara, Trail to mines detrital pools in forest leg. Short, Isaacs, Salisbury 2.xi.2013; GY13-1102-01A/ SEMC1271236 KUNHM-ENT [barcode label]/ HOLOTYPE *Desmopachriaimparis* Miller, 2021 [red label with black line border].” Paratypes, 3 in SEMC (1 male, 2 females) labeled same as holotype except with different specimen barcode labels [SEMC1271235, SEMC1271237, SEMC1271238] and each with “…PARATYPE *Desmopachriaimparis* Miller, 2021 [blue label with black line border].”

#### ﻿ Desmopachria impunctata sp. nov.

Taxon classificationAnimaliaColeopteraDytiscidae

504C87B0-41C5-5B09-B60F-F95FA9368BBA

http://zoobank.org/8248D6E7-C6F1-43F0-B298-704E02A9F4D4

Figures 60–62 , 79

##### Type locality.

Suriname, Sipaliwini District, Raleighfallen Nature Reserve, Voltzberg Trail, 04°40.910'N, 56°11.138'W.

##### Diagnosis.

This species includes medium-sized *Desmopachria* specimens in this group (TL = 1.8–1.9 mm). The elytra are more finely and indistinctly punctate than many species and punctures are of only a single, fine size. Also, the elytra are only slightly iridescent. The male genitalia are most similar to *Desmopachriaemarginata* (Figs 54–56), but the apical emargination in the median lobe in *Desmopachriaimpunctata* is relatively shallower and in lateral aspect the apex is distinctly hooked ventrad (Figs 60, 61). Also, specimens of *Desmopachriaimpunctata* are smaller (TL = 1.8–1.9 mm) than *Desmopachriaemarginata* (TL = 2.0–2.2 mm).

##### Description.

***Measurements.***TL = 1.8–1.9 mm, GW = 1.2–1.3 mm, PW = 1.0–1.1 mm, HW = 0.6–0.7 mm, EW = 0.4–0.5 mm, TL/GW = 1.5–1.6, HW/EW = 1.7–1.8. Body very broadly oval, laterally broadly curved, lateral margins slightly discontinuous between pronotum and elytron.

***Coloration.*** Head orange to orange yellow. Pronotum evenly yellow, in most specimens paler in color than head or elytron. Elytron evenly brownish orange, iridescent, especially laterally and apically. Ventral surface of head, prosternum, head appendages, and pro- and mesolegs yellow, other ventral surfaces and metalegs orange.

***Sculpture and structure.*** Head broad, short; anterior margin of clypeus broadly curved, flattened, finely margined with conspicuous, continuous narrow bead; surface of head shiny, extremely finely and sparsely punctate; eyes moderately large (HW/EW = 1.7–1.8); antennae short, scape and pedicel relatively large and rounded, flagellomere III long and slender, apically expanded, antennomeres IV-X short and broad, antennomere XI elongate, apically pointed. Pronotum short, lateral margins short, slightly curved with continuous marginal bead; surface shiny, extremely finely and sparsely punctate, posterior margin sinuate. Elytron broad, laterally broadly curved and rounded; surface shiny, extremely minutely punctate, punctures of even size, somewhat denser along elytral suture. Prosternum extremely short, longitudinally compressed, medially carinate; prosternal process in male very slender anteriorly, with low, indistinct medial tubercle, bifid apically with deep medial pit, in female slender anteriorly, with distinctive, small medial tubercle, apically short and broad, medially slightly carinate, apically acutely pointed. Metaventrite broad and evenly smoothly convex medially, surface shiny, impunctate medially, shallowly and minutely punctate laterally and posteromedially; metaventrite wings extremely slender. Metacoxa with medial portion short, < 1/2 length of metaventrite medially, metacoxal lines slightly sinuate, divergent anteriorly; lateral portion of metacoxa extremely large, anteriorly strongly expanded; surface shiny, very finely punctate. Metatrochanter very large, subequal to length of ventral margin of metafemur; legs otherwise not noticeably modified. Abdomen with surfaces shiny and smooth, very finely and sparsely punctate.

***Male genitalia.*** Male median bilaterally symmetrical, in lateral aspect elongate, evenly broad, evenly and broadly curved on both dorsal and ventral margins, apex slightly curved ventrad, pointed (Fig. 60); in ventral aspect moderately broad basally, apically expanded, lateral margins broadly rounded, apex broad, medially moderately deeply emarginate, apical branches each apically broadly triangular (Fig. 61). Lateral lobe very broad medially, irregularly and broadly narrowed to pointed apex, with series of long setae along apicodorsal margin (Fig. 62).

***Sexual dimorphism.*** Male pro- and mesotarsomeres I-III slightly more broadly expanded and with ventral adhesive setae. Male and female prosternal processes different as in all *Desmopachriaportmanni* group species.

***Variation.*** There is variation in intensity and degree of coloration across specimens. Some specimens are more evenly colored, some have greater disparity between the pale color of the pronotum and the head and elytron. Other specific variation was not detected.

##### Etymology.

This species is named *impunctata*, Latin for not punctate, for the relatively less punctate dorsal surface than in many species in the group.

##### Distribution.

This species is known from localities in Sipaliwini District, Suriname and Bolívar State, Venezuela (Fig. 79).

##### Type material.

Holotype in NZCS, male labeled, “SURINAME: Sipaliwini District 04°40.910'N, 56°11.138'W, 78 m Raleighfallen [sic] Nature Reserve Voltzberg trail; detrital pools along stream; leg. A. Short, C. McIntosh 30.vii.2012; SR12-0730-01B/ SEMC1113574 KUNHM-ENT [barcode label]/ HOLOTYPE *Desmopachriaimpunctata* Miller, 2021 [red label with black line border].” Paratypes, 232 specimens in MIZA, MSBA NCZS, and SEMC each with paratype label, “…/ PARATYPE *Desmopachriaimpunctata* Miller, 2021 [blue label with black line border]” and different barcode labels (Table 1); 23 labeled same as holotype; 24 in NZCS and SEMC labeled, “SURINAME: Sipaliwini District 3°53.942'N, 56°10.849'W, 733m CSNR: Tafelberg Summit, nr Caiman Creek Camp, pools in forest; leg. Short & Bloom 19.viii.2013; SR13-0819-05B/…”; 15 in SEMC labeled, “SURINAME: Sipaliwini District 3°53.942'N, 56°10.849'W, 733m CSNR: Tafelberg Summit, nr Caiman Creek Camp, forest detrital pools; leg. Short & Bloom 19.viii.2013; SR13-0819-05C/…”; 11 in SEMC labeled, “SURINAME: Sipaliwini District 3°53.942'N, 56°10.849'W, 733m CSNR: Tafelberg Summit, nr Caiman Creek Camp, small streams with lots of plants & leaf litter; leg. Short & Bloom 18.viii.2013; SR13-0818-03A/…”; 1 in SEMC labeled, “SURINAME: Sipaliwini District 3°53.359'N, 56°10.052'W, 879m CSNR: Tafelberg Summit, near South Rim, pool in rock leg. Short & Bloom; 20.viii.2013 SR13-0820-01C/…”; 5 in SEMC labeled, “SURINAME: Sipaliwini District 04°40.910'N, 56°11.138'W, 78 m Raleighfallen [sic] Nature Reserve Voltzberg trail; margin of stream leg. C. Maier, V. Kadosoe 30.vii.2012; sr12-0730-01A/…”; 69 in NZCA and SEMC labeled, “SURINAME: Sipaliwini District 3°53.942'N, 56°10.849'W, 733m CSNR: Tafelberg Summit, nr. Caiman Crk. Camp; dipnetting by stream margins; leg. Bloom 20.viii.2013, SR13-0820-02A/…”; 3 in SEMC labeled, “SURINAME: Sipaliwini District 3°53.942'N, 56°10.849'W, 733m CSNR: Tafelberg Summit, near Caiman Creek Camp, forest detrital pools; leg. Short & Bloom 19.viii.2013; SR13-0819-05C/…”; 69 in SEMC labeled, “SURINAME: Sipaliwini District 3°53.942'N, 56°10.849'W, 733m CSNR: Tafelberg Summit, nr Caiman Creek Camp, pools in forest; leg. Short & Bloom 19.viii.2013; SR13-0819-05B/…”; 3 in SEMC labeled, “SURINAME: Sipaliwini District 3°53.942'N, 56°10.849'W, 733m CSNR: Tafelberg Summit, near Caiman Creek Camp, pooled up detrital forest stream leg. Short & Bloom; 19.viii.2013 SR13-0819-05A/…”; 4 in SEMC labeled, “SURINAME: Sipaliwini District 3°55.600'N, 56°11.300'W, 600m CSNR: Tafelberg Summit, nr Augustus Creek Camp, pond on trail into Arrowhead basin leg. Short & Bloom; 16.viii.2013 SR13-0816-02A/…”; 1 in SEMC labeled, “SURINAME: Sipaliwini District 3°55.600'N, 56°11.300'W, 600m CSNR: Tafelberg Summit, nr Augustus Creek Camp, pools & creeks on trail into Arrowhead basin; leg. Short & Bloom 17.viii.2013; SR13-0817-01A/…”; 3 in SEMC labeled, “SURINAME: Sipaliwini District 3°53.942'N, 56°10.849'W, 733m CSNR: Tafelberg Summit, near Caiman Creek Camp, stream margins: legs. Short & Bloom 18.viii.2013; SR13-0818-01A/…”; 1 in MIZA labeled, “VENEZUELA: Bolívar State 6°35.617'N, 66°49.238'W, 80m Los Pijiguaos: outcrop/morichal 12.i.2009; leg Miller & Short VZ09-0112-01C: detrial [sic] pools/…”

#### ﻿ Desmopachria truncata sp. nov.

Taxon classificationAnimaliaColeopteraDytiscidae

20305740-00A4-5BE8-87CF-E3CBEB6D4754

http://zoobank.org/E50C2B82-C005-4A50-8044-5842D61074C1

Figures 63–65 , 81

##### Type locality.

Suriname, Sipaliwini District, Camp 4, Kasikasima, stream on trail to METS camp, 200m, 2.97731°N 55.38500°W.

##### Diagnosis.

This species is characterized by the male median lobe slender, apically distinctly expanded with the apex broadly truncate and the lateral lobe longer than the median lobe (Figs 64, 65). The dorsal punctation on the head, pronotum and elytra is very fine and sparse. The elytra are iridescent. The genitalia are somewhat similar to *Desmopachriamutata* Sharp, but that species has the median lobe very slender, not as broadly expanded apically, and apically rounded, not truncate (Young 1995: fig. 2). That species also occurs in Mexico, not northern South America (Young 1995). *Desmopachriatruncata* is otherwise not similar to other species in the *Desmopachriaportmanni-aldessa* subgroup.

##### Description.

***Measurements.***TL = 2.0–2.1 mm, GW = 1.3–1.4 mm, PW = 1.0–1.1 mm, HW = 0.6–0.7 mm, EW = 0.3–0.4 mm, TL/GW = 1.4–1.5, HW/EW =1.8–2.2. Body very broad, laterally rounded, lateral margins continuous between pronotum and elytron; dorsoventrally compressed.

***Coloration.*** Head and pronotum evenly dark orange, head slightly iridescent dorsally. Elytron evenly dark orange, iridescent, especially apically. Ventral surface of head, prosternum, head appendages, and pro- and mesolegs yellow, other ventral surfaces and metalegs darker orange, lateral portion of metacoxa and abdominal ventrites somewhat iridescent.

***Sculpture and structure.*** Head broad, short; anterior margin of clypeus finely margined with continuous flattened narrow bead; surface of head shiny, punctation extremely fine, sparse but evenly distributed; eyes moderately large (HW/EW = 1.8–22); antennae short, scape and pedicel relatively large and rounded, flagellomere III long and slender, apically expanded, antennomeres IV-X short and broad, antennomere XI elongate, apically pointed. Pronotum short, lateral margins short, broadly curved with continuous narrow bead; surface shiny, punctation very fine, irregular, sparse; posterior margin sinuate. Elytron broad, laterally broadly curved; surface shiny, punctate, punctures dual, fine and large interspersed, without linear series. Prosternum extremely short, longitudinally compressed, medially slightly carinate; prosternal process in male very slender anteriorly, with low, indistinct medial tubercle, bifid apically with deep medial pit, in female slender anteriorly, with distinctive, small medial tubercle, apically short and broad, medially slightly carinate, apically acutely pointed. Metaventrite broad and evenly smoothly convex medially, surface shiny, impunctate; metaventrite wings extremely slender. Metacoxa with medial portion short, < 1/3 length of metaventrite medially, metacoxal lines sinuate, divergent anteriorly; lateral portion of metacoxa extremely large, anteriorly strongly expanded; surface shiny, impunctate. Metatrochanter very large, subequal to length of ventral margin of metafemur; legs otherwise not noticeably modified. Abdomen with surfaces shiny and smooth, very finely and sparsely punctate.

***Male genitalia.*** Male median lobe in lateral aspect elongate, evenly curved, apically narrowly rounded (Fig. 63; in ventral aspect elongate, evenly constricted medially, expanded apically to abruptly broadly truncate apex (Fig. 64). Lateral lobe extending distinctly beyond apex of median lobe, moderately broad, apically rounded with dense margin of long setae apically and along apicodorsal margin (Fig. 65).

***Sexual dimorphism.*** Male pro- and mesotarsomeres I-III slightly more broadly expanded and with ventral adhesive setae. Male and female prosternal processes different as in all *Desmopachriaportmanni* group species.

***Variation.*** The single specimen from “Upper Palumeu” has the male median lobe slightly longer and more slender with the lateral lobes apically a little more rounded. This does not seem to represent a significant difference however, and the specimen is from the same general area as the holotype. The series from Guyana similarly has some shape variation in the lateral and median lobes, but again, it does not seem to suggest species-level differences. However, additional specimens could help clarify the limits in these populations. A couple specimens are paler tan in color, but this could be because they are teneral.

##### Etymology.

This species is named *truncata*, Latin for the characteristic apically truncate male median lobe in this species.

##### Distribution.

This species is known from Sipaliwini District, Suriname and Region VIII, Guyana (Fig. 81).

##### Type material.

Holotype in NZCS, male labeled, “SURINAME: Sipaliwini District 2.97731°N, 55.38500°W 200 m Camp 4 (low, Kasikasima; sandy stream on trail to METS camp 20.iii.2012; SR12-0320-02A leg. A. Short; 2012 CI-RAP Survey/ SEMC1087011 KUNHM-ENT [barcode label]/ HOLOTYPE *Desmopachriatruncata* Miller, 2021 [red label with black line border].” Paratypes, 17; 14 in NZCS and SEMC labeled same as holotype except each with different barcode labels (Table 1); 1 in SEMC labeled, “SURINAME: Sipaliwini District N 2.47700', W 55.62941', 275 m Camp 1, Upper Palumeu leg. A. Short; Flight Intercept Trap 10-16.iii.2012; SR12-0310-TN1 2012 CI-RAP Survey/ SEMC1089093 KUNHM-ENT; 2 in CBDG and SEMC labeled, “GUYANA: Region XIII [sic] 5°10.514'N, 59°28.970'W, 440 m Kaiteur Nat’l Park, trail by guest house; small forest stream leg. A. Short; 15.iii.2014 GY14-0315-03A/ SEMC1328988 KUNHM-ENT [barcode label]” and “…SEMC1328993 KUNHM-ENT].” All paratypes also with “…PARATYPE *Desmopachriatruncata* Miller, 2021 [blue label with black line border].”

###### *Desmopachriaportmanni-aldessa* subgroup – iridescent *Desmopachria* with a forked male prosternal process (= Desmopachria (Pachiridis) Young, 1980)

*Desmopachriaaldessa* Young, 1980 – Brazil (Braga and Ferreira-Jr. 2014), Trinidad

*Desmopachriaanastomosa* sp. nov. – Guyana

*Desmopachriaangulata* sp. nov. – Guyana, Suriname

*Desmopachriaaurea* Young, 1980 – Brazil (Braga and Ferreira-Jr. 2014), Suriname

*Desmopachriaemarginata* sp. nov. – Suriname

*Desmopachriaimparis* sp. nov. – Guyana

*Desmopachriaimpunctata* sp. nov. – Suriname

*Desmopachriairidis* Young, 1980 – Brazil

*Desmopachrianovacula* Young, 1980 – Suriname

*Desmopachriatruncata* sp. nov. – Guyana, Suriname.

### ﻿The *Desmopachriaportmanni-portmanni* subgroup

**Diagnosis.** Within the *Desmopachriaportmanni* species-group, these species have the dorsal surface not iridescent.

**Comments.** These species tend to be either dorsally distinctly maculate or evenly darkly colored, but all without iridescence, but males have a distinctive bifid prosternal process with a medial pit as with all *Desmopachriaportmanni*-species.

#### ﻿ Desmopachria bisulcata sp. nov.

Taxon classificationAnimaliaColeopteraDytiscidae

F4A9B5E0-F1E4-525E-862E-E776AF77F196

http://zoobank.org/2F6FFF06-FD08-423A-97E4-890185DAB3DA

Figures 66–68 , 81

##### Type locality.

Suriname, Sipaliwini District, Camp 3, Werehpai, SE Kwamala, 2°22.259'N 56°41.227'W, 229m.

##### Diagnosis.

This species has a relatively simple median lobe that is slender, elongate, and curved in lateral aspect, and broad basally and apically evenly narrowed to a narrowly rounded apex in ventral aspect (Figs 66, 67). The lateral lobe is small and slender in lateral aspect, and much shorter than the median lobe (Fig. 68). The dorsal sculpture is shiny and with dual punctation in most specimens. No specimens of *Desmopachriabisulcata* display iridescence. In some specimens of both males and females the dorsum is matte, with a microreticulation that obscures the punctation. Other species with similar male median lobe shapes are *Desmopachriairidis* and *Desmopachriaanastomosis*, but specimens of each of these are dorsally iridescent, whereas those of *Desmopachriabisulcata* are not.

##### Description.

***Measurements.***TL = 2.0–2.1 mm, GW = 1.4–1.5 mm, PW = 1.0–1.1 mm, HW = 0.7–0.8 mm, EW = 0.4–0.5 mm, TL/GW = 1.4, HW/EW = 1.9–2.0. Body very broad, laterally rounded, lateral margins continuous between pronotum and elytron; dorsoventrally compressed.

***Coloration.*** Head and pronotum evenly orange-red, same ***coloration.*** Elytron evenly brownish orange, not iridescent. Ventral surfaces evenly orange-red.

***Sculpture and structure.*** Head broad, short; anterior margin of clypeus finely margined with continuous flattened narrow bead; surface of head shiny, but matte; eyes large (HW/EW = 1.9–2.0); antennae short, scape and pedicel relatively large and rounded, flagellomere III long and slender, apically expanded, antennomeres IV-X short and broad, antennomere XI elongate, apically pointed. Pronotum short, lateral margins short, broadly curved with continuous narrow bead; surface matte to shiny, but punctation very fine, of the same size and evenly distributed, posterior margin sinuate. Elytron broad, laterally broadly curved; surface matte or, less commonly, shiny; punctation very fine, of the same size and evenly distributed across most of elytron, when punctate, dual with a few minute, interspersed punctures laterally. Prosternum extremely short, longitudinally compressed, medially slightly carinate; prosternal process in male very slender anteriorly, with low, indistinct medial tubercle, bifid apically with deep medial pit, in female slender anteriorly, with distinctive, small medial tubercle, apically short and broad, medially slightly carinate, apically acutely pointed. Metaventrite broad and evenly smoothly convex medially, surface matte, finely and irregularly punctate laterally; metaventrite wings extremely slender. Metacoxa with medial portion short, < 1/3 length of metaventrite medially, metacoxal lines sinuate, divergent anteriorly; lateral portion of metacoxa extremely large, anteriorly strongly expanded; surface matte, finely, evenly punctate, not iridescent. Metatrochanter very large, subequal to length of ventral margin of metafemur; legs otherwise not noticeably modified. Abdomen with surfaces shiny and smooth, very finely and sparsely punctate.

***Male genitalia.*** Male median lobe in lateral aspect slender, evenly curved on both ventral and dorsal margins to narrowly pointed apex (Fig. 66); in ventral aspect very broad basally, evenly narrowed to broadly rounded apex (Fig. 67). Lateral lobe small, slender, apically slender, and slightly hooked dorsally with dense margin of long setae (Fig. 68).

***Sexual dimorphism.*** Male pro- and mesotarsomeres I-III slightly more broadly expanded and with ventral adhesive setae. Male and female prosternal processes different as in all *Desmopachriaportmanni* group species. Males tend to be both shinier and more punctate than females with are matte and not punctate in the few specimens examined.

***Variation.*** Some specimens are matte between punctures on elytra. This does not seem correlated with sex, males and females may both be either shiny or matte. Extent of punctation seems somewhat variable among the sexes.

##### Etymology.

This species is named *bisulcata*, Latin for the male forked prosternal process.

##### Distribution.

This species is known from one locality in Sipaliwini District, Suriname (Fig. 81).

##### Type material.

Holotype in NZCS, male labeled, “SURINAME: Sipaliwini District 02°22.259'N, 56°41.227'W, 229m Camp 3; Werehpai, SE Kwamala detrital pools in dense forest 3-5.ix.2010;leg. Short & Kadosoe CI-rap Survey; SR10-0903-02A/ SEMC0912211 KUNHM-ENT [barcode label]/ HOLOTYPE *Desmopachriabisulcata* Miller, 2021 [red label with black line border].” Paratypes, 15 in NZCS, MSBA, and SEMC labeled same as holotype except with different specimen barcode labels (Table 1) and each with “…PARATYPE *Desmopachriabisulcata* Miller, 2021 [blue label with black line border].”

#### ﻿ Desmopachria irregulara sp. nov.

Taxon classificationAnimaliaColeopteraDytiscidae

F182407B-F917-5965-A4C8-C010C564271F

http://zoobank.org/58E77BB3-EC2F-4F6A-85E7-E0FCBEF2E331

Figures 69–71 , 81

##### Type locality.

Venezuela, Zulia State, Perija National Park, Tukuko, Río Manantial, 9°50.490'N, 72°49.310'W.

##### Diagnosis.

This species is distinctive for the non-iridescent dorsal and ventral surfaces and the characteristic male median and lateral lobes. The median lobe has an extremely large lobe on the dorsal surface of the median lobe medially (Fig. 69). The lateral lobe is broad throughout with a short, dense series of setae along the apical and subapical dorsal margins and a cluster of elongate setae subapically on the ventral margin (Fig. 71). The elytral punctation is large with few minute punctures interspersed. The pronotal punctation is extremely fine except posteromedially where it is large and dense.

##### Description.

***Measurements.***TL = 2.1–2.2 mm, GW = 1.4–1.5 mm, PW = 1.1–1.2 mm, HW = 0.7–0.8 mm, EW = 0.4 mm, TL/GW = 1.4–1.5, HW/EW = 1.8–1.9. Body very broad, laterally rounded, lateral margins continuous between pronotum and elytron; dorsoventrally compressed.

***Coloration.*** Head and pronotum evenly orange-red. Elytron evenly orange-red, similar to pronotum and head, not iridescent. Metaventrites, metacoxae and abdominal ventrites orange-red, other surfaces orange.

***Sculpture and structure.*** Head broad, short; anterior margin of clypeus finely margined with continuous flattened narrow bead; surface of head shiny, impunctate medially, finely punctate posteriorly; eyes large (HW/EW = 1.8–1.9); antennae short, scape and pedicel relatively large and rounded, flagellomere III long and slender, apically expanded, antennomeres IV-X short and broad, antennomere XI elongate, apically pointed. Pronotum short, lateral margins short, shallowly curved with continuous narrow bead; surface shiny, punctation very fine and sparse over most of surface, punctation large and dense posteromedially, posterior margin sinuate. Elytron broad, laterally broadly curved; surface shiny, punctation dual, mostly large, relatively dense, with fewer fine punctures interspersed. Prosternum extremely short, longitudinally compressed, medially slightly carinate; prosternal process in male very slender anteriorly, with low, indistinct medial tubercle, bifid apically with deep medial pit, in female slender anteriorly, with distinctive, small medial tubercle, apically short and broad, medially slightly carinate, apically acutely pointed. Metaventrite broad and evenly smoothly convex medially, surface shiny, impunctate; metaventrite wings extremely slender. Metacoxa with medial portion short, < 1/3 length of metaventrite medially, metacoxal lines sinuate, divergent anteriorly; lateral portion of metacoxa extremely large, anteriorly strongly expanded; surface shiny, very finely punctate, not iridescent. Metatrochanter very large, subequal to length of ventral margin of metafemur; male metafemur curved ventrally with conspicuous series of short setae long entire margin. Abdomen with surfaces shiny and smooth, finely punctate.

***Male genitalia.*** Male median lobe in lateral aspect irregular, ventral margin abruptly expanded submedially, evenly convexly curved in apical 2/5, ventral margin with large, apically subtruncate lobe medially, apex slight broadened, apex subtruncate (Fig. 69); in ventral aspect slender, lateral margins shallowly sinuate, apex deeply bifid, lateral rami apically broad, directed slightly mesad (Fig. 70). Lateral lobe very broad throughout length, slightly curved dorsad, apex broadly rounded, subapically on ventral surface with small cluster of elongate setae, apex and subapically on dorsal margin with dense series of short setae (Fig. 71).

***Sexual dimorphism.*** Only males were examined, but male pro- and mesotarsomeres I-III appear to be slightly more broadly expanded and with ventral adhesive setae, and male prosternal process is different as in all *Desmopachriaportmanni* group species.

***Variation.*** There is little variation between the two male specimens examined.

##### Etymology.

This species is named *irregulara*, Latin for irregular, for the unusual shape of the male median lobe.

##### Distribution.

This species is known from one locality in Zulia State, Venezuela (Fig. 81).

##### Type material.

Holotype in MIZA, male labeled, “VENEZUELA: Zulia State 9°50.490'N, 72°49.310'W, Perija, Nat. Park: Tukuko: Río Manatial 29.i.2009; Short, García, Camacho VZ09-0129-01B: detrital pool/ SM0845453 KUNHM-ENT [barcode label]/ HOLOTYPE *Desmopachriairregulara* Miller, 2021 [red label with black line border].” Paratypes, 1 in SEMC labeled same as holotype except with “…SM08454561… [barcode label]” and with “…PARATYPE *Desmopachriairregulara* Miller, 2021 [blue label with black line border].”

###### Checklist of *Desmopachriaportmanni-portmanni* subgroup – includes the non-iridescent *Desmopachria* with forked male prosternal process (= Desmopachria (Portmannia) Young, 1980)

*Desmopachriabasicollis* Guignot, 1950 – Costa Rica

*Desmopachriabisulcata* sp. nov. – Suriname

*Desmopachriabryanstoni* Clark, 1862 – Mexico, Panama, Guatemala

= *Desmopachriapolita* Sharp, 1882

*Desmopachriacarranca* Braga & Ferreira‑Jr., 2018 – Brazil

*Desmopachriadecorosa* Young, 1995 – Mexico

*Desmopachriadicrophalica* Braga & Ferreira‑Jr., 2014 – Brazil

*Desmopachriadispar* Sharp, 1882 – Mexico

*Desmopachriadisticta* Braga & Ferreira‑Jr., 2014 – Brazil

*Desmopachriaduodentata* Braga & Ferreira‑Jr., 2011 – Brazil

*Desmopachriagoias* Young, 1995 – Brazil

*Desmopachriagrammosticta* Braga & Ferreira‑Jr., 2014 – Brazil

*Desmopachriagrandinigra* Braga & Ferreira‑Jr., 2014 – Brazil

*Desmopachriairregulara* sp. nov. – Venezuela

*Desmopachriaitamontensis* Braga & Ferreira‑Jr., 2014 – Brazil

*Desmopachrialaevis* Sharp, 1882 – Brazil (Braga and Ferreira-Jr. 2014)

*Desmopachriamutata* Sharp, 1882 – Brazil

*nomen novum* for *Desmopachriabryanstoni* Sharp, 1882

*Desmopachrianiger* Zimmermann, 1923 – Brazil (São Paulo

*Desmopachrianigricoxa* Braga & Ferreira‑Jr., 2018 – Brazil

*Desmopachrianigrisphera* Braga & Ferreira‑Jr., 2018 – Brazil

*Desmopachrianitidissima* Zimmermann, 1928 – Brazil

*Desmopachriapittieri*Young 1995 – Costa Rica

*Desmopachriaportmanni* Clark, 1862 – USA and N Mexico

*Desmopachriasobrina* Young, 1995 – Panama

*Desmopachriaspecula* Sharp, 1887 – Panama (probably not part of *Desmopachriaportmanni* species group according to Young (1995)).

*Desmopachriaukuki* Braga & Ferreira‑Jr., 2014 – Brazil

*Desmopachriaundulatosterna* Braga & Ferreira‑Jr., 2011 – Brazil

*Desmopachriavariegata* Sharp, 1882 – Mexico, El Salvador, Honduras

*Desmopachriayoungi* Miller, 1999 – Bolivia

*Desmopachriazetha* Young, 1995 – Mexico

### ﻿*Desmopachriastriola* species group

**Diagnosis.** This species group is characterized by the elytron with a sutural stria (Miller 2001; Miller and Wolfe 2019; Young 1980, 1990b).

**Comments.**Miller and Wolfe (2019) described new species and discussed the likelihood that the group is not monophyletic since the sutural stria is not a particularly reliable synapomorphy in Dytiscidae and some of the species in this group are not similar in other ways. Within the group are several species, though, that have similar genitalia with the median lobe “shouldered” medially in ventral aspect and the lateral lobe characteristically angled in lateral aspect. The following new species appears to belong to this subgroup of the *Desmopachriastriola* species group.

#### ﻿ Desmopachria robusta sp. nov.

Taxon classificationAnimaliaColeopteraDytiscidae

F1871AC2-06A8-5CED-AE08-1A6FA6776EC7

http://zoobank.org/AA30C940-1C89-42EB-98AD-4484BC305A57

Figures 72–75 , 81

##### Type locality.

Venezuela, Zulia State, Perija National Park, Tukuko, Rio Manantial, 9°50.490'N, 72°49.310'W.

##### Diagnosis.

This species is similar to other species in the *Desmopachriastriola* species group with “shouldered” median lobes (Miller and Wolfe 2019), but the male median lobe in this species is considerably broader than in other species (Fig. 74).

##### Description.

**Figures 72–75.**
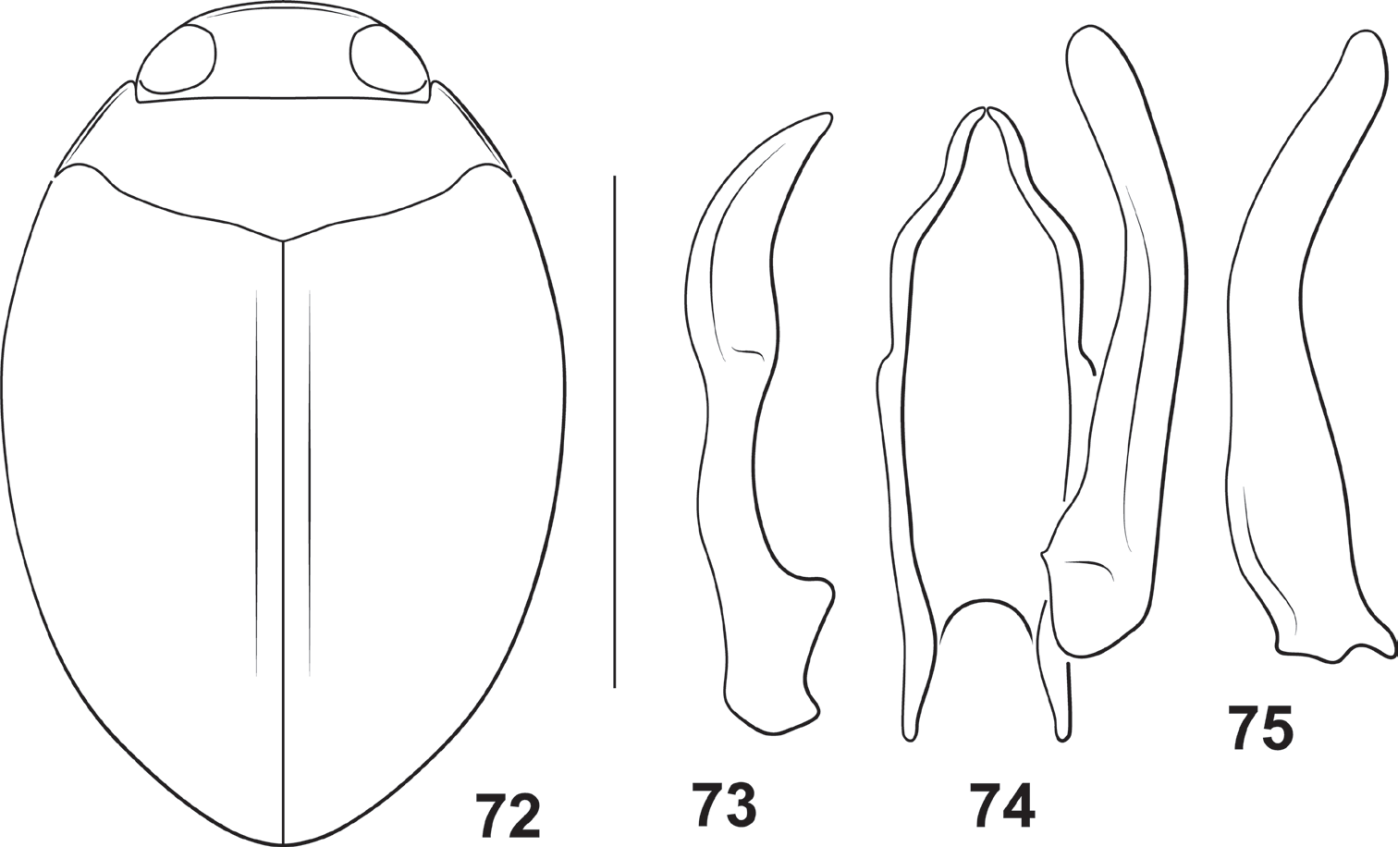
*Desmopachriarobusta*. **72** habitus **73** lateral lobe, right lateral aspect **74** median lobe and right lateral lobe, ventral aspect **75** right lateral lobe, right lateral aspect. Scale bar: 1.0 mm.

***Measurements.***TL = 1.7 mm, GW = 1.1 mm, PW = 0.8 mm, HW = 0.6 mm, EW = 0.3 mm, TL/GW = 1.5, HW/EW = 2.2. Body very broad, broadly rounded, laterally broadly curved, lateral margins continuous between pronotum and elytron, body broadest across elytra at midlength of body (Fig. 72).

***Coloration.*** Dorsal surface of head and pronotum evenly yellow. Elytron pale orange-brown, narrowly darker along anterior and sutural margins. Head appendages, legs, and ventral surfaces orange-yellow.

***Sculpture and structure.*** Head broad, anteriorly curved; anterior margin of clypeus curved, flattened, margined with conspicuous, continuous narrow bead; surface of head shiny, finely and sparsely punctate; eyes large (Fig. 72, HW/EW = 2.2); antennae short, scape and pedicel relatively large and rounded, flagellomere III long and slender, apically expanded, antennomeres IV-X short and broad, lobed at anterodorsal angle, antennomere XI elongate, apically pointed. Pronotum very short, lateral margins short, slightly curved with continuous narrow bead, slightly wider medially; surface shiny, impunctate medially, very finely and indistinctly punctate around margins. Elytron moderately broad, laterally broadly curved; surface shiny, extremely finely punctate across surface; with distinctive subsutural stria, or groove, extending most of length of elytron (Fig. 72). Prosternum extremely short, longitudinally compressed, medially slightly carinate; prosternal process slender anteriorly, with distinctive, small medial tubercle, apical blade short and broad, medially concave, apically broadly pointed. Metaventrite broad and evenly smoothly convex medially, surface shiny, impunctate; metaventrite wings extremely slender. Metacoxa with medial portion short, < 1/3 length of metaventrite medially, metacoxal lines divergent anteriorly, sinuate; lateral portion of metacoxa extremely large, anteriorly strongly expanded; surface shiny, impunctate. Metatrochanter large, subequal to length of ventral margin of metafemur; legs otherwise not noticeably modified. Abdomen with surfaces shiny and smooth, impunctate.

***Male genitalia.*** Male median lobe elongated in lateral aspect, robust, apical half broad, broadly and evenly curved dorsad to narrowly rounded apex (Fig. 73); in ventral aspect very broad, robust, lateral margins subparallel basally, with two distinct “shoulders” one medially, and one more distinct subapically, apically distinctly narrowed with medial rounded apex (Fig. 74). Lateral lobe in lateral aspect moderately throughout length, distinctly bent dorsad, apex slightly curved ventrad, rounded (Fig. 75); in ventral aspect evenly broad throughout length, medially distinctly bent mediad (Fig. 74).

***Sexual dimorphism and variation.*** Only a male specimen was examined.

**Figures 76, 77.**
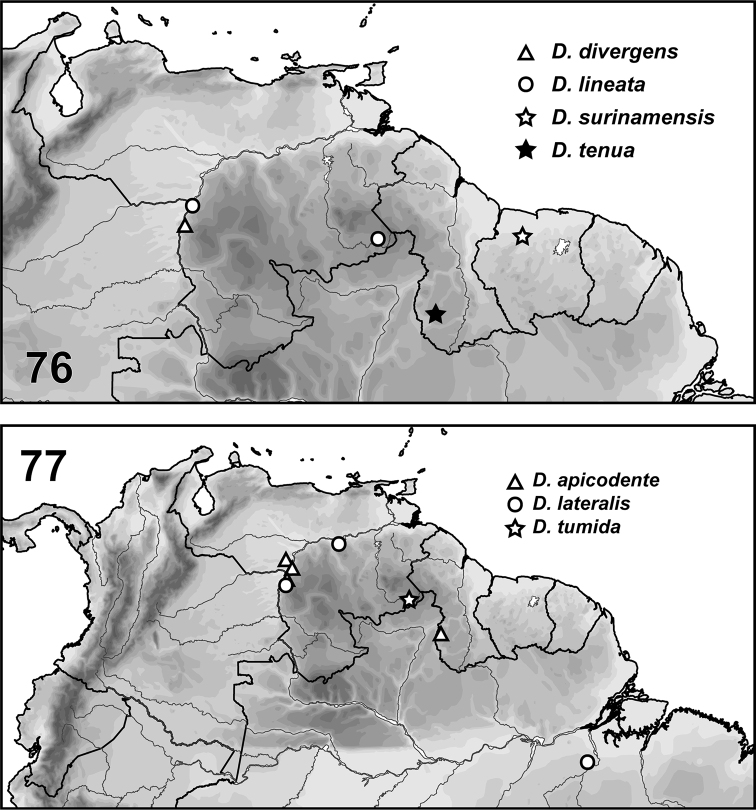
*Desmopachria* species distributions. **76** ungrouped *Desmopachria* species **77***Desmopachriaapicodente* species group.

##### Etymology.

This species is named *robusta*, Latin for strong or robust, for the conspicuously broader male median lobe than that of other similar species in the genus.

##### Distribution.

This species is known from Zulia State, Venezuela (Fig. 81).

##### Habitat.

The type specimen was collected from a “detrital pool.”

##### Type material.

Holotype in MIZA, male labeled, “VENEZUELA: Zulia State 9°50.490'N, 72°49.310'W, Perija Nat. Park: Tukuko: Rio Manantial 29.i.2009; Short, García, Camacho VZ09-0129-01B: detrial [sic] pool/ SM0844647 KUNHM-ENT [barcode label]/ HOLOTYPE *Desmopachriarobusta* Miller, 2021 [red label with black line border].”

**Figure 78.**
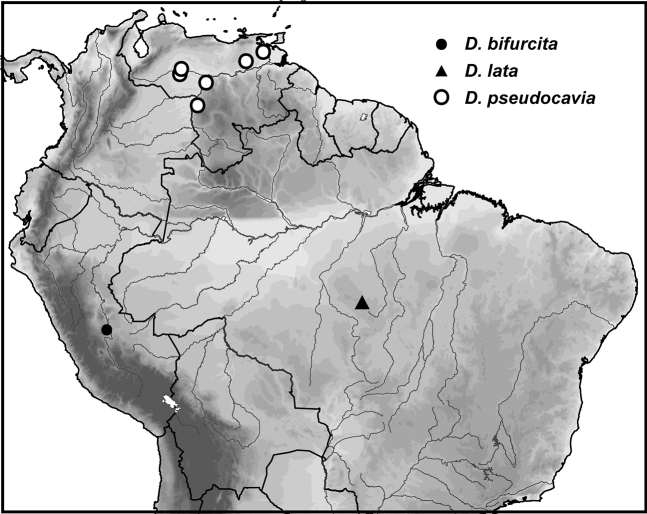
*Desmopachria* species distributions. *Desmopachriabifurcita* species group and *Desmopachriaconvexa* species group.

###### Checklist of *Desmopachriastriola* species group

*Desmopachriaamyae* Miller, 2001 – Bolivia, Brazil (Braga and Ferreira-Jr. 2014)

*Desmopachriaatropos* Miller & Wolfe, 2019 – Venezuela

*Desmopachriachei* Miller, 1999 – Bolivia

*Desmopachriachlotho* Miller & Wolfe, 2019 – Suriname

*Desmopachriaferrugata* Régimbart, 1895 – Brazil

*Desmopachriafossulata* Zimmermann, 1928 – Brazil (Braga and Ferreira-Jr. 2014)

*Desmopachriagrouvellei* Régimbart, 1895 – Mexico, Argentina, Paraguay?

*Desmopachrialachesis* Miller & Wolfe, 2019 – Guyana, Suriname, Venezuela

*Desmopachriarobusta* sp. nov. – Venezuela

*Desmopachriaruginosa* Young, 1990 – Brazil

*Desmopachriastriola* Sharp, 1887 – Argentina, Bolivia, Brazil (Braga and Ferreira-Jr. 2010), Colombia, Costa Rica, Ecuador, Guatemala, Panama, Peru, Suriname, Trinidad, USA (Florida), Venezuela.

### ﻿*Desmopachriavicina* species group

**Diagnosis.** This group is characterized by the anterior metatibial spine serrate (Miller 2001) (historically the subgenus D. (Nectoserrula) Guignot, 1949 (Guignot 1949; Young 1980).

**Figures 79, 80.**
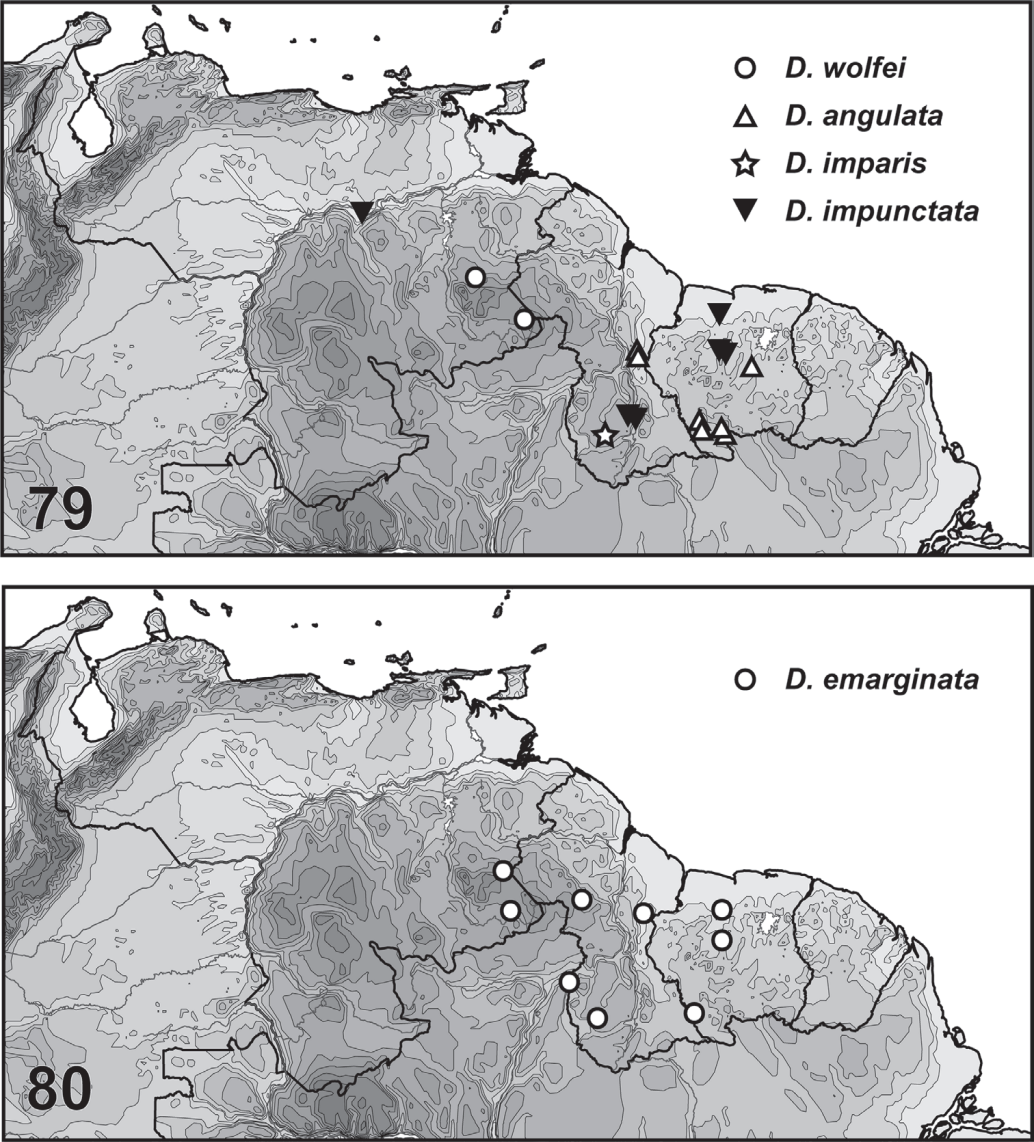
*Desmopachria* species distributions. **79***Desmopachrianitida* species group and *Desmopachriaportmanni-aldessa* species group **80***Desmopachriaportmanni-aldessa* species group.

**Comments.** No new species have been discovered in this group for over 100 years.

#### Checklist of *Desmopachriavicina* species group

*Desmopachriaconcolor* Sharp, 1882 – Uruguay

*Desmopachriamendozana* (Steinheil, 1869) – Argentina.

*Desmopachriapunctatissima* Zimmermann, 1923 – Argentina

*Desmopachriavicina* Sharp, 1887 – Mexico

**Figure 81.**
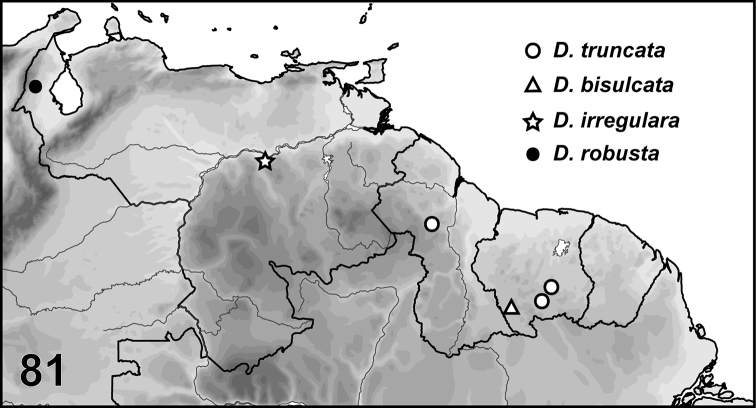
*Desmopachria* species distributions, *Desmopachriaportmanni-aldessa* species group, *Desmopachriaportmanni-portmanni* species group, and *Desmopachriastriola* species group.

### ﻿*Desmopachriaubangoides* species group

**Diagnosis.** These are iridescent *Desmopachria* without a forked male prosternum and with the anterior clypeal margin dimorphic, more developed in male (= Desmopachria (Hintonia) Young, 1980 (Guignot 1949; Young 1980)

**Comments.** A new species in this group, *D.yanomami* Braga & Ferreria Jr., 2018, was described recently.

#### Checklist of *Desmopachriaubangoides* species group

*Desmopachriaubangoides* Young, 1980 – Brazil, Ecuador

*Desmopachriasiolii* Young, 1980 – Brazil

*Desmopachriaminuta* Young, 1980 – Brazil

*Desmopachriayanomami* Braga & Ferreira Jr., 2018 – Brazil, Venezuela (new record, Venezuela, Amazonas State, Communidad Caño Gato on Rio Sipapo, 4°58.838'N, 67°44.341'W, MIZA, SEMC, MSBA).

### ﻿*Desmopachriaglabricula* species group

**Diagnosis.** These are iridescent *Desmopachria* without a forked male prosternum and with the anterior clypeal margin dimorphic and more developed in males (= Desmopachria (Hintonia) Young, 1980 (Guignot 1949; Young 1980)

**Comments.** New species have been described recently in this group (Braga and Ferreira-Jr. 2014; Miller 1999, 2001).

#### Checklist of *Desmopachriaglabricula* species group

*Desmopachriaaphronoscelus* Miller, 1999 – Bolivia

*Desmopachriaflavida* Young, 1981 – Mexico.

*Desmopachriaglabricula* Sharp, 1882 – Guatemala.

*Desmopachrialeechi* Young, 1981 – USA, Florida.

*Desmopachriastethothrix* Braga & Ferreira Jr., 2014 – Brazil.

*Desmopachriastrigata* Young, 1981 – Brazil.

*Desmopachriavolatidisca* Miller, 2001 – Bolivia.

*Desmopachriavolvata* Young, 1981– Panama.

*Desmopachriazimmermani* Young, 1981 – Mexico.

## Supplementary Material

XML Treatment for Desmopachria divergens

XML Treatment for Desmopachria lineata

XML Treatment for Desmopachria surinamensis

XML Treatment for Desmopachria tenua

XML Treatment for Desmopachria apicodente

XML Treatment for Desmopachria lateralis

XML Treatment for Desmopachria tumida

XML Treatment for Desmopachria bifurcita

XML Treatment for Desmopachria lata

XML Treatment for Desmopachria pseudocavia

XML Treatment for Desmopachria wolfei

XML Treatment for Desmopachria angulata

XML Treatment for Desmopachria emarginata

XML Treatment for Desmopachria imparis

XML Treatment for Desmopachria impunctata

XML Treatment for Desmopachria truncata

XML Treatment for Desmopachria bisulcata

XML Treatment for Desmopachria irregulara

XML Treatment for Desmopachria robusta
